# Transcriptomics in lung tissue upon respiratory syncytial virus infection reveals aging as important modulator of immune activation and matrix maintenance

**DOI:** 10.1038/s41598-018-35180-2

**Published:** 2018-11-09

**Authors:** Jeroen L. A. Pennings, Rob Mariman, Hennie M. Hodemaekers, Sylvia S. N. Reemers, Riny Janssen, Teun Guichelaar

**Affiliations:** 1 Centre for Health Protection, National Institute for Public Health and the Environment (RIVM), Bilthoven, The Netherlands; 2 Centre for Infectious Disease Control, National Institute for Public Health and the Environment (RIVM), Bilthoven, The Netherlands; 3 Present Address: MSD Animal Health, Boxmeer, The Netherlands

## Abstract

Aging poses an increased risk of severe infection by respiratory syncytial virus (RSV). The many different biological pathways comprising the response to infection in lungs that are influenced by aging are complex and remain to be defined more thoroughly. Towards finding new directions in research on aging, we aimed to define biological pathways in the acute response to RSV that are affected in the lungs by aging. We therefore profiled the full transcriptome of lung tissue of mice prior to and during RSV infection both at young and old age. In the absence of RSV, we found aging to downregulate genes that are involved in constitution of the extracellular matrix. Moreover, uninfected old mice showed elevated expression of pathways that resemble injury, metabolic aberrations, and disorders mediated by functions of the immune system that were induced at young age only by an exogenous trigger like RSV. Furthermore, infection by RSV mounted stronger activation of anti-viral type-I interferon pathways at old age. Despite such exaggerated anti-viral responses, old mice showed reduced control of virus. Altogether, our findings emphasize important roles in aging-related susceptibility to respiratory disease for extracellular matrix dysfunctions and dysregulated immune activation in lungs.

## Introduction

Old age poses an increased risk for severe disease of the airways caused by respiratory syncytial virus (RSV). In the elderly population it has been estimated that numbers of hospitalization due to RSV infection may reach close to the numbers of respiratory illness caused by influenza viruses^1–3^. In mice, RSV has been shown to cause more lung pathology at old age, but the different mechanisms comprising the response to infection that are influenced by aging of the lung tissue largely remain to be defined. Studies on the full transcriptome in young adult mice have shown that the response to RSV at the site of infection is complex as it involves many different biological pathways^4,5^. Altered antiviral expression of some genes in lung due to aging^6,7^ and increased susceptibility to infectious diseases indicate the involvement of unique age-related features that should be considered towards understanding disease at old age. However, effects of aging on the full RSV-induced transcription profile in lungs have remained elusive. In our study, we determined the full transcription profile in the lungs during infection with RSV to provide a more comprehensive exploration of the consequences of old age on gene expression.

Increased susceptibility to disease caused by RSV in the elderly has largely been ascribed to age-related decline of the levels of protective antibodies and memory function of cells of the adaptive immune system resulting in insufficient RSV-specific immunity^8–11^. Infection due to this lack of acquired RSV-specific immunity implies a prominent role for direct innate anti-viral immune mechanisms that are activated in infected tissue in order to control the virus. A prominent role for innate responses has been exemplified by a study that shows genetic susceptibility to RSV-induced bronchiolitis in children being predominantly associated with innate immune genes^12^. Moreover, these innate genes comprise pro-inflammatory genes, indicating a prominent role for inflammatory conditions in susceptibility to disease by RSV-infection^12^. Hence, innate immune responses to RSV do not only control the infection but can also induce inflammation that contributes to pathology and is associated with RSV bronchiolitis^7,12^. The importance of the acute responses of the innate immune compartment in disease is also underscored by studies in blood of RSV-challenged human adults. These showed that many of the genes that discriminate symptomatic individuals from asymptomatic individuals are involved in innate and acute immune responses induced by viruses^13,14^. Altogether, these findings suggest an important role for the acute anti-viral response and its resulting inflammation during RSV infection at old age.

The expression of some pro-inflammatory cytokines produced in lungs in response to RSV infection and clearance of virus have been reported to be delayed at old age in cotton rat and mouse models^6–8^. These observations with respect to RSV are in line with the general view that many of the innate responses that participate in defeating pathogens become dysfunctional at old age^15,16^. Paradoxically, although inflammatory responses that are induced via these innate immune mechanisms may be compromised at old age, aging is accompanied by chronically increased levels of markers of inflammation, a phenomenon that is known as inflammaging^17^. Such chronic inflammation at old age has been observed in lungs of mice in the absence of specified pathogens^18^. Despite the plethora of studies on inflammaging and aging-related alterations of immune responses to RSV, the biological pathways of the acute response to RSV *in situ* in the lungs that are affected by aging are still elusive.

To define the biological pathways in the acute response to RSV that are affected by old age, we determined the full transcription profiles of lung tissue of adult mice in the primary response to RSV-infection at young and old age. Comparing these primary responses to RSV at young and old age, as well as between uninfected mice of both age groups, provide insights into age-related deficiencies in processes that pose increased susceptibility to RSV. Moreover, genes and pathways identified by this approach will point towards previously neglected biological mechanisms that may be key to RSV-induced pneumonia at old age and direct future research on aging-related susceptibility to infectious respiratory diseases.

The BALB/c model is widely used to study responses to RSV *in vivo* since it reflects the antigen-specific Th2-biased pathology in FI-vaccinated children and BALB/c mice have shown relative susceptibility to infection with human RSV. According to some studies, this mouse strain is somewhat more permissive to RSV replication than the C57BL/6 mice that are often used to study cellular immunity to intracellular antigens^19,20^. Nonetheless, C57BL/6 mice are well suited for studying RSV replication, acute disease and immune responses resulting from RSV infection^19,21,22^. Several studies have shown that levels of replication and disease by human RSV in C57BL/6 mice can get similar to the levels found in BALB/c mice^21,22^. We explored the impact of aging in C57BL/6 mice, since mice of this strain are widely utilized to study anti-viral cellular responses and provide the background for many transgenic and knockout mice used to study immune responses^19,23^.

## Results

**Figure 1:**
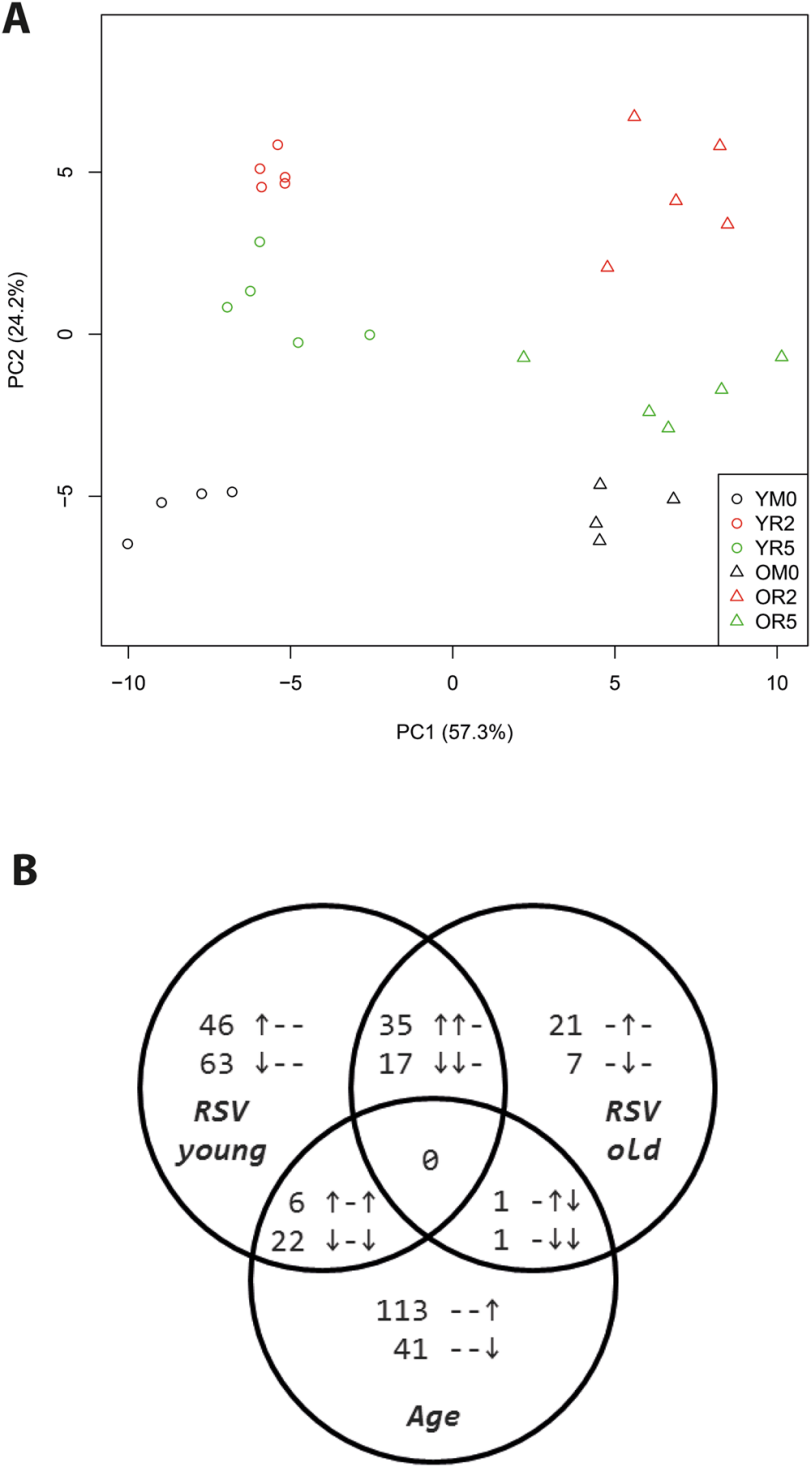
Distinct effects by aging and RSV on gene expression. Panel A shows each individual animal in a principal component analysis (PCA) of the microarray data of lung samples derived at day 0 (mock-inoculated, black, n = 4/group), or at day 2 (red, n = 5/group) or day 5 (green, n = 5/group) after inoculation with RSV. Aging-regulated expression appears by PC1, RSV-regulated gene expression appears along PC2. Y, young (circles); O, old (triangles); M, mock control; R, RSV-inoculated. Panel B shows a Venn-diagram depicting numbers of genes that are significantly upregulated (↑), downregulated (↓), or show no significant response (−). The three symbols indicate from left to right: response to RSV in young mice, response to RSV in old mice, response to aging, respectively.

We quantified transcription in lung tissue of young and old mice at 2 and 5 days after inoculation with RSV, and in lung tissue of non-inoculated young and old mice. For the analysis of RSV-regulated genes we considered the genes that were significantly up or downregulated at any of the two time points upon inoculation. We analysed dissimilarities between the 6 groups for the combined set of all regulated genes by principal component analysis (PCA) (Fig. 1A). This showed a clear separation between all groups. The first principal component (PC1) explains 57% of all variance and shows differences between age groups under all conditions. Moreover, as illustrated by the Venn diagram in Fig. 1B, the young and old groups show both unique and shared up and downregulated genes (Fig. 1B).

**Figure 2:**
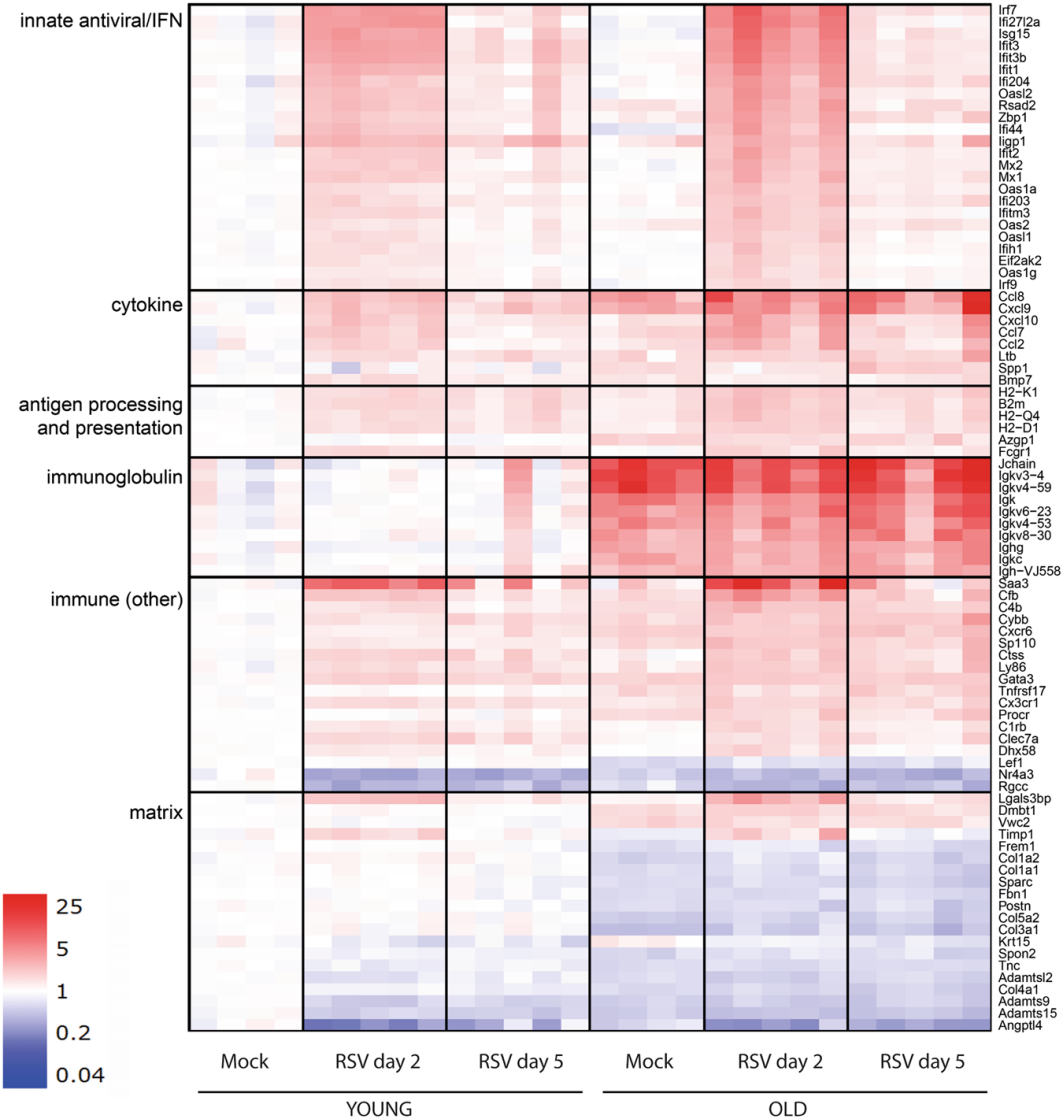
Heatmap showing differentially regulated genes. Map shows significantly upregulated (red) and downregulated (blue) genes at day 0 (mock-inoculated), or at day 2 or 5 after inoculation with RSV for all individual mice at young or old age, categorized by function.

Functional annotation analysis by DAVID^24^ of all 373 significantly regulated genes under any of the conditions by age and/or response to RSV at any of the days after inoculation with RSV revealed that several pathways and functionalities were overrepresented, which –for further analysis– we grouped into several umbrella categories. We categorized genes with known immunological functions as innate interferon (IFN)-mediated antiviral function (24 genes), cytokines (8 genes), antigen processing and presentation (6 genes), immunoglobulins (10 genes), or other immune function (18 genes). Also, twenty genes involved in matrix constitution were significantly regulated in our study. For the six categories mentioned above, different expression patterns can be recognized (Fig. 2). In addition, 172 genes with known functionalities were annotated by functions other than these aforementioned categories. Of all differentially regulated genes, 70 genes were not annotated as having a function (‘unknown function’) and 45 genes are only known as gene models based on their sequence, but it is not known if they correspond to actual genes or have a functionality. For our further analyses of genes annotated by DAVID, we included only the genes that could be categorized by the six functional categories.

### Aging regulates gene expression in lungs under basal conditions

Aging itself induces alterations in expression of a considerable number of genes as shown by comparison of lungs of non-RSV inoculated mice at young and old age (Fig. 1B). Notably, 15 percent of these genes that were regulated by aging in the absence of RSV infection were also significantly regulated by RSV in young mice (Fig. 1B), indicating that aging induces biological features in lungs that are found at young age only upon exposure to RSV. Thus, aging of lung tissue defines a distinctive set of altered gene expressions in steady state.

In the absence of RSV we observed in lungs of old mice a major elevation of expression of immunoglobulin genes (*J chain*, *Igkv3-4*, *Igkv4-59*, *Igk*, *Igkv6-23*, *Igkc*, *Ighg*, *Igh-VJ558*), compared to lungs of young mice (Table 1). Some of these, among which the *J chain*, showed the highest upregulation of all genes regulated in the study. Among the genes we categorized further we observed elevated expression at old age for the cytokine genes *Cxcl9* and *Spp1*, for a gene involved in antigen processing and presentation (*Azgp1*), for eight other immune-related genes (*Cxcr6*, *Gata3*, *Sp110*, *Procr*, *Cybb*, *Tnfrsf17*, *C4b*, *Cfb*), and for two matrix-related genes (*Vwc2* and *Dmbt1*). Among the gene expression that was downregulated by aging we found many genes that are involved in matrix constitution (*Col3a1*, *Adamts15*, *Adamts9*, *Spon2*, *Col4a1*, *Tnc*, *Fbn1*, *Col1a1*, *Postn*, *Adamtsl2*, *Sparc*, *Frem1*), but only one gene that could be classified by immune function (*Lef1*). These data indicate that aging leads to elevated expression of immunological activity in lungs, and downregulation of genes involved in constitution of the extracellular matrix of lung tissue prior to infection.

**Table 1:** Gene expression affected by age.

Upregulated genes	Fold change	Function	Downregulated genes	Fold change	Function
Jchain	11.85	immunoglobulin	Col3a1	−2.32	matrix
Igkv3-4	11.55	immunoglobulin	Adamts15	−1.91	matrix
Igkv4-59	11.13	immunoglobulin	Adamts9	−1.82	matrix
Igk	5.53	immunoglobulin	Spon2	−1.67	matrix
Igkv6-23	3.97	immunoglobulin	Col4a1	−1.63	matrix
Igkc	3.11	immunoglobulin	Tnc	−1.63	matrix
Ighg	2.85	immunoglobulin	Fbn1	−1.61	matrix
Igh-VJ558	2.46	immunoglobulin	Col1a1	−1.59	matrix
Cxcl9	3.02	cytokine	Postn	−1.58	matrix
Spp1	1.60	cytokine	Adamtsl2	−1.57	matrix
Cxcr6	1.84	immune (other)	Sparc	−1.55	matrix
Gata3	1.79	immune (other)	Frem1	−1.52	matrix
Sp110	1.63	immune (other)	Lef1	−1.64	immune (other)
Procr	1.62	immune (other)			
Cybb	1.61	immune (other)			
Tnfrsf17	1.60	immune (other)			
C4b	1.59	immune (other)			
Cfb	1.56	immune (other)			
Azgp1	1.78	antigen proc&pres			
Vwc2	1.68	matrix			
Dmbt1	1.57	matrix			

Positive values depict fold higher expression, negative values depict fold reduction in uninfected old mice compared to uninfected young mice.

Antigen proc&pres; antigen processing and presentation.

Immune (other) indicates genes related to immune-related categories other than innate antiviral interferon-mediated functions, antigen processing and presentation, immunoglobulins, or cytokines.

### Response to RSV in lungs distinctive of young age

The principal component analysis shows differences between groups related to the response to RSV by principle component 2 (PC2). Young and old mice showed a response to RSV roughly in the same direction and with a similar overall magnitude. Overall, in both young and old animals the fold change of gene expression in response to RSV was strongest at two days after virus inoculation (Fig. 1A). RSV regulated 109 genes in young mice that were not found to be significantly altered in RSV-inoculated lungs at old age (Fig. 1B). As shown in Table 2, we found upregulated expression of cytokine genes (*Ccl8*, *Cxcl10*, *Ccl7*, *Ccl2*, *Ltb*, *Bmp7*), genes involved in antigen processing and presentation (*B2m*, *H2-K1*, *Fcgr1*, *H2-D1*, *H2-Q4*), one gene of the innate antiviral IFN-pathway (*Iigp1*), and genes involved in other immune functions (*Saa3*, *Ctss*, *Clec7a*, *Cx3cr1*, *Ly86*). Among the downregulated genes, we found one gene involved in matrix (*Angptl4*) and two genes with immune functions (*Nr4a3*, *Rgcc*). All other downregulated genes were annotated by other functionalities than immune or matrix functions (Supplemental Table S1). Some responses to RSV were found also at old age (e.g. induction of B2M and other molecules involved in antigen processing and presentation) or were even stronger (e.g. *Saa3*, *Cxcl10*) in old mice but did not reach significance. These data indicate that aging causes loss of responsiveness in different molecular pathways involved in the innate immune response to RSV in the lungs.

**Table 2:** Gene expression affected by RSV at young age only.

Upregulated genes	day 2	day 5	Function	Downregulated genes	day 2	day 5	Function
Saa3	7.52	2.35	immune (other)	Angptl4	−4.67	−2.31	matrix
Ctss	1.79	1.60	immune (other)	Nr4a3	−3.14	−2.96	immune (other)
Clec7a	1.66	1.60	immune (other)	Rgcc	−2.70	−2.37	immune (other)
Cx3cr1	1.58	1.51	immune (other)				
Ly86	1.56	1.52	immune (other)				
Ccl8	2.35	1.72	cytokine				
Cxcl10	2.03	1.37	cytokine				
Ccl7	1.95	1.25	cytokine				
Ccl2	1.80	1.14	cytokine				
Ltb	1.53	1.56	cytokine				
Bmp7	1.51	1.27	cytokine				
Iigp1	2.52	2.14	innate antiviral/IFN				
B2m	1.68	1.55	antigen proc&pres				
H2-K1	1.62	1.58	antigen proc&pres				
Fcgr1	1.61	1.29	antigen proc&pres				
H2-D1	1.55	1.43	antigen proc&pres				
H2-Q4	1.54	1.44	antigen proc&pres				

Positive values depict fold higher expression, negative values depict fold reduction in RSV-infected mice compared to young uninfected mice.

Notably, of the genes that are regulated by aging only (i.e. without RSV infection), some were also significantly regulated by RSV in young mice (Table 3), indicating that aging induces biological features in lungs that are found at young age only upon exposure to RSV. Among the downregulated genes we found three adamts-encoding genes. Moreover, expressions of a set of genes involved in immune functions that are induced in response to RSV at young age were significantly elevated at old age (*Cxcl9*, *Cfb*, *C4b*, *Cybb*, *Gata3*). This indicates enhanced immunological activity in the lungs at old age that would be induced at young age only by an extrinsic trigger like an infection by RSV. This suggests partial anti-viral immune activity that may be involved in protection against infection at the time of inoculation with RSV particularly at old age.

**Table 3:** Gene expression affected by RSV and by aging.

Upregulated genes	Aging	Response to RSV		
		day 2	day 5	Function
Cxcl9	3.02	2.13	1.88	cytokine
Cfb	1.56	2.15	1.36	immune (other)
C4b	1.59	1.52	1.20	immune (other)
Cybb	1.61	1.44	1.62	immune (other)
Gata3	1.79	1.80	1.68	immune (other)
**Downregulated genes**	**Aging**	**Response to RSV**		
		**day 2**	**day 5**	**Function**
Adamts15	−1.91	−1.79	−1.89	matrix
Adamts9	−1.82	−1.86	−1.69	matrix
Adamtsl2	−1.57	−1.48	−1.23	matrix

Positive values depict fold higher expression, negative values depict fold reduction by aging or by response to RSV in young mice.

### Gene expression regulated by RSV in the context of aging

Overall, the principal component analysis indicates that variation of responsiveness to RSV between individuals is larger in the old age group compared to the young RSV-inoculated mice (Fig. 1A). This larger variation was also reflected in the pooled standard deviation in gene expression within each group, and shows more intragroup heterogeneity in old mice two and five days after inoculation with RSV (Table 4). Moreover, a unique set of genes was upregulated by RSV exclusively at old age (Table 5). Most of the upregulated immune-related genes in the RSV inoculated old animals were found in the antiviral IFN-pathway (*Ifi204*, *Oas1a*, *Oas2*, *Eif2ak2*, *Oas1g*) and only two in other immune pathways (*C1rb*, *Dhx58*). Some genes, like *Plxna2* and *Klfl1* were downregulated in response to RSV exclusively at old age, but none of these genes were within the six selected categories. These data show that aging promotes responsiveness to RSV of a unique set of genes that are not significantly activated by RSV at young age, and a substantial part of these genes are involved in innate antiviral IFN-pathways.

**Table 4:** Relative standard deviation.

	No	Day 2	Day 5
	infection	post-infection	post-infection
Young	13.26	11.93	18.31
Old	13.33	19.60	23.98

Numbers show relative standard deviation as a percentage of the mean expression per gene within each group.

**Table 5:** Gene expression affected by RSV at old age only.

Upregulated genes	day 2	day 5	Function
C1rb	1.66	1.28	immune (other)
Dhx58	1.61	1.10	immune (other)
Ifi204	3.14	1.59	innate antiviral/IFN
Oas1a	2.09	1.27	innate antiviral/IFN
Oas2	1.99	1.42	innate antiviral/IFN
Eif2ak2	1.70	1.04	innate antiviral/IFN
Oas1g	1.68	1.16	innate antiviral/IFN

Values depict fold higher expression in old RSV-infected mice compared to uninfected old mice.

Our further analyses defined a unique set of genes that were up and down regulated in response to RSV at both young and old age (Table 6). Notably, of all molecules related to immune responsiveness within this unique set, only molecules that are part of the innate antiviral IFN pathway were involved. Expressions of these antiviral molecules were all upregulated and comprised a major part of all RSV regulated genes. Moreover, most of these molecules of the innate antiviral IFN pathway were activated more strongly at old age. Altogether, the data indicate that RSV induces a stronger activation of the local innate anti-viral IFN pathway at old age.

**Table 6:** Gene expression affected by RSV at young and old age.

Regulated genes	Young		Old		Function
	day 2	day 5	day 2	day 5	
Irf7	3.74	1.50	5.63	1.49	innate antiviral/IFN
Ifi27l2a	3.88	1.25	5.56	1.22	innate antiviral/IFN
Isg15	3.49	1.50	4.78	1.40	innate antiviral/IFN
Ifit3	3.43	1.74	4.47	1.41	innate antiviral/IFN
Ifit3b	3.22	1.72	4.18	1.40	innate antiviral/IFN
Ifit1	2.69	1.43	3.56	1.41	innate antiviral/IFN
Oasl2	2.19	1.40	3.05	1.25	innate antiviral/IFN
Rsad2	2.19	1.46	3.03	1.39	innate antiviral/IFN
Zbp1	1.96	1.39	2.86	1.68	innate antiviral/IFN
Ifi44	2.14	1.37	2.68	1.05	innate antiviral/IFN
Ifit2	1.96	1.29	2.50	1.29	innate antiviral/IFN
Mx2	1.91	1.19	2.39	1.27	innate antiviral/IFN
Mx1	1.97	1.36	2.38	1.35	innate antiviral/IFN
Ifi203	1.62	1.32	2.05	1.48	innate antiviral/IFN
Ifitm3	1.68	1.14	2.01	1.12	innate antiviral/IFN
Oasl1	1.52	1.15	1.91	1.13	innate antiviral/IFN
Ifih1	1.51	1.14	1.79	1.14	innate antiviral/IFN
Irf9	1.46	1.16	1.58	1.04	innate antiviral/IFN
Lgals3bp	2.26	1.29	3.02	1.40	matrix
Timp1	1.72	1.06	1.86	−1.17	matrix

Positive values depict fold higher expression, negative values depict fold reduction.

### Viral loads in lungs show impaired clearance of RSV due to aging

**Figure 3:**
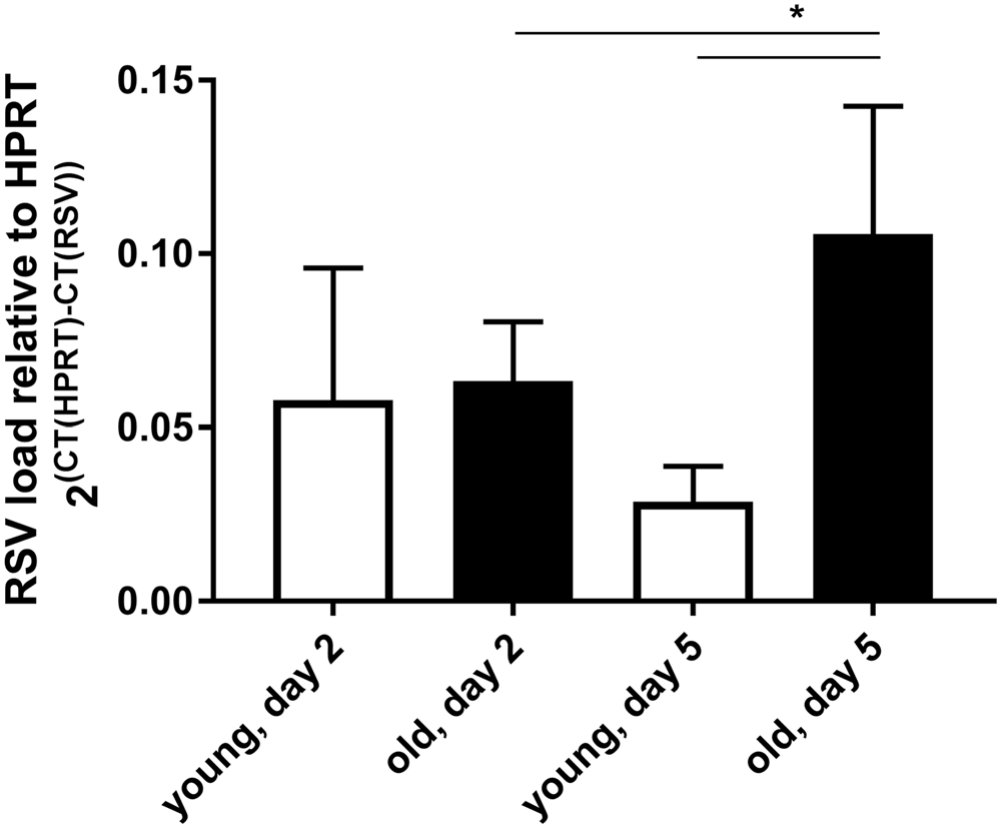
RSV detected in lungs by RT Q-PCR. RSV load was quantified by RT Q-PCR on viral RNA and depicted as amount of viral RNA relative to the amount of RNA encoding the housekeeping gene HPRT detected by RT Q-PCR in the same specimen. The amount of RSV RNA was quantified by calculating the threshold cycle values (C_T_) for RSV relative to the C_T_ values measured for HPRT using the 2^ΔCT^ method. Figure shows expression in lungs at day 2 or day 5 after inoculation with RSV in young and old mice. RSV was not detected in RT Q-PCR on mock-inoculated lungs. *P < 0.05 (ANOVA).

Among the RNA isolated from lungs at two and five days after inoculation with RSV we analysed the amount of RSV-transcripts by qPCR to determine clearance of the virus over the period in which we performed our arrays (Fig. 3). The amount of RSV relative to housekeeping gene *Hprt* was similar between young and old mice at two days after inoculation, whereas in mock controls no RSV was detected (Ct > 40). This indicates that viral loads in lungs did not differ between young and old mice at two days after inoculation. However, viral loads significantly increased further until day five only in the old group, leading to significantly higher viral loads in old mice compared to young mice. This indicates that the first line of defence of aged mice functions less adequately to control a respiratory infection with RSV.

### Validation of microarray data

For ten significantly regulated genes, selected from five pathways or functions, we performed Q-PCR validation (Supplemental Table S3). Overall, the extent of regulation found by Q-PCR correlated well with that found by microarray analysis (Spearman R = 0.92). The magnitude of the up- or down-regulation was somewhat larger in the Q-PCR data, with the median difference to the corresponding microarray values being 17%. This slightly enhanced effect size in Q-PCR is in agreement with previous findings^4^. In functional terms, the Q-PCR analyses confirmed the main findings of the microarray analyses, namely: stronger activation of anti‐viral type‐I interferon pathways in old mice than in young mice; down-regulation by age of genes involved in extracellular matrix; up-regulation of cytokines by old age as well as due to RSV infection; as well as the expression pattern found for antigen processing and presentation and other immune functionality.

### Predicted upstream regulators and disease-related pathways indicate functional interference between aging and anti-viral responses

Next, we aimed to predict which upstream molecules that regulate the expression of genes we analysed may be affected by aging. Considering the genes that were significantly regulated in our arrays as downstream effectors, we applied Ingenuity to predict which molecules may serve as their upstream regulators and are affected by aging. Table 7 shows regulators for which an activated or inhibited status was predicted by cognate enrichment (quantifying change by z-score > 2 and P < 1 × 10^−10^) for the combined up and downregulation of the expression of the downstream molecules we found altered significantly in our arrays. This analysis predicted inhibition of a set of upstream regulators by aging under steady state conditions (dexamethasone, IL4, Vegf, TGFB1, NR3C1, AGT). These regulatory processes influenced by aging point at defective immune suppression via glucocorticoid receptors (dexamethasone and the glucocorticoid receptor NR3C1) and TGF-B.

**Table 7:** Predicted inhibition of upstream regulators by aging (p < 10^−10^).

Aging	Status	z-score
*dexamethasone*	Inhibited	−3.18
*IL4*	Inhibited	−2.72
*Vegf*	Inhibited	−2.62
*TGFB1*	Inhibited	−2.47
*NR3C1*	Inhibited	−2.29
*AGT*	Inhibited	−2.22

Aging and infection by RSV are both known to involve biological processes that are part of disease. We questioned whether the profile of gene expression in the lungs that is altered by aging may indicate overlap with known disease profiles. To this end, we analysed enrichment by significantly regulated genes in our study for known biological disorders by Ingenuity (Table 8). This analysis showed that the expression profile induced by RSV infection in the lungs showed most resemblance with the same five diseases in young and old mice. Among these biological disorders, we found RSV-unrelated diseases like gastrointestinal disease, endocrine system disorders, and metabolic disorders. However, even in the absence of an RSV infection we found that aging enriched for some of these diseases that were enriched for in response to RSV, i.e. organismal injury (136 genes), immunological disease (55 molecules), and metabolic disease (49 genes). In addition, the transcriptomic profile of aging in lung tissue resembled processes involved in cancer (134 genes) and haematological disease (55 genes). These findings suggest that aging induces biological alterations in lungs that resemble injury, involve aberrations of metabolism, and disorders mediated by functions of the immune system that would be induced at young age only by exogenous pathogens like RSV.

**Table 8:** Top diseases and biological functions.

Aging	^#^Molecules
Organismal Injury and Abnormalities	136
Cancer	134
Hematological Disease	55
Immunological Disease	55
Metabolic Disease	49
**RSV young**	^**#**^ **Molecules**
Organismal Injury and Abnormalities	151
Gastrointestinal Disease	145
Immunological Disease	84
Metabolic Disease	54
Endocrine System Disorders	53
**RSV old**	^**#**^ **Molecules**
Organismal Injury and Abnormalities	54
Immunological Disease	39
Gastrointestinal Disease	34
Endocrine System Disorders	24
Metabolic Disease	24

## Discussion

Our study aimed to point out molecules and previously neglected biological mechanisms in lung tissue that may aid in directing the focus of future research on aging-related susceptibility to infectious respiratory diseases. Our findings show that aging shapes alteration of the expression of unique sets of genes in lung tissue in both steady state and during a response to infection with RSV. Prior to challenge with virus, aged lungs showed reduced expression of genes involved in extracellular matrix function and showed differential expression of genes indicative of uncontrolled elevation of immunological activity and injury, cancer, immunological disease and metabolic disease. Moreover, the first line of defence in RSV-infected lungs controls the virus less effectively at old age, whereas RSV appeared to induce excessive immune responses by the antiviral type-I IFN pathways in aged lungs. Alternatively, although RSV-induced interferon responses were higher at old age and some of the elevated immune activities at old age prior to infection are part of the anti-viral response, these immune mediators could not effectively control RSV. These findings indicate that aging may interfere with the immune homeostatic regulation in a way that leaves the immune system refractory to RSV and prohibits clearing of virus. Towards development of preventive and therapeutic interventions, this warrants directing the focus of further research on susceptibility to infectious respiratory diseases at old age to the role of dysregulated inflammation in viral pathogenesis and the role of altered extracellular matrix functions in susceptibility to infection and the physics of lung disease during infection.

Studies on gene expression in lung tissue under steady state condition and upon infection with RSV have indicated a wide range of aging-related alterations among broad panels of immune-related genes^6,7^. By analysing the full transcriptome, we found many other immune-relate genes and also many non-immune genes can be added to the spectrum of gene expressions impacted by aging, both under steady state conditions and upon inoculation with RSV. A substantial number of the genes we found being expressed at lower levels at old age are involved in constituting or remodelling of the extracellular matrix, such as genes encoding collagens and periostin (*Postn*). Moreover, our analyses indicated enrichment of gene expression changes resembling injury in the aged lungs under homeostasis. Collagens sustain the functional architecture of healthy lung tissue as they provide a major constituent of the extracellular matrix scaffold on which cells in the lung tissue perform their functions and which impacts the mechanical forces involved in breathing^25^. Periostin is involved in airway remodelling as it promotes wound repair by epithelial cells and it can also induce collagen I and III gene expression^26^. The matrix scaffolds the epithelial cells that are targeted by RSV and provides the environment needed by immune cells and antiviral molecules to optimally migrate in the infected lungs. Moreover, remodelling of the extracellular matrix is needed for the physical constitution needed to breathe optimally and to provide an optimal barrier function preventing entry of pathogens. When the walls of alveoli are destroyed, the air sacs coalesce, which results in airflow obstruction^25^ as found in chronic obstructive pulmonary disease (COPD)^27^. Aging impacts the breathing capacity of lungs and the morphology and elasticity of the lungs^28–31^, but most studies on aging of lung tissue address defective matrix functions in the context of non-infectious diseases such as COPD. The need of the extracellular matrix for immune cells to locally control infection and the effect of physical obstructions caused by RSV that may add to the reduced respiratory capacity in aged lungs are often neglected in studies on aging-related infectious diseases of the lung tissue. Downregulated matrix remodelling was a major part of all aging-regulated changes of gene expression and therefore indicates that, in addition to altered immune responsiveness, a dysfunctional matrix may have a major impact on setting the stage for increased susceptibility to disease by infection at old age. We cannot exclude that the downregulation of matrix genes may actually be an underestimation due to transcripts being diluted by genes expressed by cells of the immune system invading the lungs at old age^18,32^. Nonetheless, our data warrant more detailed studies on the role of dysfunctional remodelling of the lung matrix contributing to susceptibility to aging-related respiratory disease in the context of pathogenic infections.

The high expression level of immunoglobulin genes combined with the high expression of the joining chain (J chain) of multimeric antibodies IgA and IgM, suggests the abundance of memory B cells or plasma cells producing IgM and mucosal IgA antibodies at old age. Indeed, in lavages of human bronchia elevated levels of IgA and IgM have been found at old age indicating increase of antibody producing cells in the lungs by aging^29^. This may be due to accumulation of memory B cells residing in lung tissue upon infections^33^ experienced over the years. Another explanation may be found in the increased immune activity represented in a set of genes in our study and found by others in elevated numbers of pulmonary lymphocytes at old age and in low-grade chronic inflammation, known as inflammaging^18,29,34^. These conditions are likely to attract immune cells into the aged lung tissue. Moreover, our analyses suggested downregulation of immunosuppressive upstream mechanisms associated with molecules like TGFB1, glucocorticoid receptor NR3C1 and Dexamethasone. This finding indicates defective responsiveness to anti-inflammatory pathways that are naturally exerted by our immune system to prevent immune-mediated diseases may be responsible for the elevated activation status of immune cells in aged lung tissue. Since inflammatory conditions are involved in many aging-related diseases and frailty, it would be interesting to find out in future research to what extent inflammaging of the lung tissue may contribute to aging-related susceptibility to respiratory infections. Understanding of how elevated immune activities in lung tissue may contribute to dysfunctions and disease will provide targets that can be used to design treatments and preventive measures other than vaccines alone.

Aged lungs mounted an abundant response to RSV in innate anti-viral interferon pathways. Many genes of these pathways were even expressed to a higher level than in RSV-inoculated lungs at young age. Type I interferons are key to the direct innate response to a viral infection that is required to prevent further infection and spread of viruses. Since old mice less effectively control the viral burden while they highly expressed genes involved in anti-viral interferon pathways, our findings indicate that the anti-viral interferon pathways at old age may function less effectively and may be enhanced by higher viral loads due to dysfunctions in other anti-viral functionalities at old age. Additional analyses by Ingenuity of prediction of upstream regulators indicated defective natural downregulation of STAT3 upon RSV infection at old age (Supplemental Table S2). Since STAT3 has been shown to negatively regulate the inflammatory properties of type I IFNs^35^, suggesting that inadequate control of the interferon pathway leads to abundant activation of interferon pathways by infections at old age. Alternatively, dysregulation of the interferon pathways by aging may also be found in the very early kinetics of response to virus. Several studies have shown that innate immune cells from lung mount a slower innate interferon response to virus within the first hours upon encounter of virus. Our analyses and studies by others focused on later time-points during responsiveness in the infected airways, but future research on the age dynamics of the interferon pathways *in situ* should clarify on this age-related phenomenon of the dynamics during the first day of a viral challenge. In addition to direct anti-viral functions, activation of the innate interferon pathways have been shown to induce inflammation that may harm the lung tissue^36^. This leads to suggest that exaggerated induction of interferon pathways by uncontrolled virus may drive inflammation that causes enhanced pathogenesis during an infection at old age. Studies have shown that anti-inflammatory treatment during RSV infection can prevent pathology even though these anti-inflammatory modulators diminish control of viral replication^7^. Altogether, these findings warrant thorough studies on dysregulation of the balance between anti-viral versus pro-inflammatory functions of interferons in disease by pulmonary infections at old age. Moreover, improving the use of anti-inflammatory interventions may be promising and gain focus over just the induction of stronger adaptive immune responses in the context of vaccination.

Induction of genes by RSV at young age that was not found at old age, indicate that aging causes loss of responsiveness in different molecular pathways involved in the immune responses to RSV in the lungs. Among these we found a set of chemokines like CXCL10 and CCL8 that regulate migration of immune cells in tissue and molecules such as FCGR1 and MHC-related genes that confer antigen processing and presentation to orchestrate specific immunity by adaptive immune cells. These processes have indeed been reported to be impaired at old age. However, since the group of old mice showed higher variation we cannot exclude that lack of statistically significant induction of all these genes at old age may be due to lower statistical power in the aged animals.

Interestingly, the inter-animal variation was comparable in uninfected young and old mice, but variation of responsiveness to RSV between individuals is larger in the old age group compared to the young RSV‐inoculated mice, indicating that the higher variation between old animals becomes apparent only upon infection with RSV. Notably, increased biological variation at old age is a major biological phenomenon that is now gaining more attention in order to better understand the processes involved in aging and hence lead to dysfunctions at old age^37–40^.

Notably, we found in lungs that aging induces expression of a set of genes that are expressed at young age only upon infection with RSV. Many of these have relevant functions in the immune system and suggest an antiviral immune status partly being active chronically, and maybe in a way that makes the immune system refractory to RSV. Expression of CXCL9, also known as MIG, a chemokine that attracts T cells and NK cells via the ligand CXCR3^41^, may explain the chronically elevated level of these cells in aged lungs reported by others. Expression of CYBβ, a microbicidal protein that is part of the oxidase system of phagocytes^42^, also indicates elevated antimicrobial activity in lungs at old age. Elevated GATA3 we found in old animals may indicate enhanced activity by type 2 T-cell responses and allergy-like lung inflammation, since GATA3 is a transcription factor that is expressed by Th2 cells and Type 2 innate lymphoid cells, and promotes susceptibility to allergy-like inflammation associated with Th2 responses^43–46^. However, Ingenuity analyses predicted downregulation of Th2-inducers or inhibition of responsiveness to these inducers (IL-4 and VEGF^47^) and therefore suggests regulation of GATA3-mediated effects other than mere propagation of Th2 functions^45^. CFB and C4b are molecules of the alternative complement pathway and known to be produced in large amounts in the liver. Finding complement expression in aged lungs and upon RSV infection indicates that the liver may not be the sole and major organ for producing complement under some settings. These findings suggest that aging induces biological alterations in functions of the immune system that all would be induced at young age only by exogenous pathogens like RSV. Whether this is a result of dysfunctional immune regulation at old age that may contribute to susceptibility or, alternatively, maybe a requirement for needed enhancement of immune patrol to retain sufficient immunity at old age remains to be defined.

Cotton rats are highly susceptible to infection and disease by RSV. They provide the most representative small animal model to study responsiveness to RSV infection, but tools that enable thorough cellular analyses and the number of published studies on aging in the cotton rat model are limited. Nonetheless, some of the key features of aging indicated by our analyses have been indicated also in this model. Our findings are in line with the limited number of studies on responsiveness to RSV in cotton rats, as these studies showed slower clearance of virus, stronger pro-inflammatory responsiveness in RSV-infected lungs, and enhanced acute responses to classical innate stimuli at old age^7,8,39,48^.

Our findings indicate multiple new directions and considerations for future research. One important consideration for future studies is the notion that RSV may infect alveolar type I and type II epithelial cells at different rates, but may also infect immune cells like alveolar macrophages^49,50^. Both the cellular composition of the lungs and the functionality of different cell subsets may be subject to aging^51,52^. Therefore, future studies should reveal which of the different types among the alveolar cells contribute most to aging-related effects we found and how molecular pathways we found to be affected by aging may mechanistically interact in these alveolar cell types. For example, mechanistic *in vitro* studies with alveolar type II epithelial cells have shown that TGF-β promotes differentiation of these cells from type II into type I cells^53^. Ingenuity analyses of our data indicated hampered TGF-β signalling at old age, suggesting that hampered TGF-β signalling may contribute to reduced transdifferentiation of alveolar cells in lungs and hence lead to altered ratio of alveolar type I and type II pneumocytes at old age. Moreover, it would be interesting to define the impact of RSV, or selected proteins derived from the virus, on regulation of inflammatory genes and extracellular matrix genes. Such future approaches based on findings like ours will open a new era in research on the impact of aging on susceptibility to RSV.

Differences between mouse strains, such as C57BL/6 we used or BALB/c strains used by others^6^, and differences between mouse and human samples may interfere with interpretations on the effect of aging on expression of selected genes of interest^54^. Nonetheless, our study indicates that research on compromised immunity to infections by aging should look beyond the defective induction and functionality of memory cells and antibodies to RSV at old age. Future studies should therefore address means that could improve primary barrier function in lungs, dysregulated innate immune responses in the lungs, and means to control exaggerated pro-inflammatory immune activity that contribute to pathology.

## Methods

### Animals

Female specific-pathogen-free (SPF) C57BL/6JRccHsd mice were obtained from Harlan Laboratories (Horst, The Netherlands). At the start of the experiment young mice were used at the age of 8 to 10 weeks, old mice were used at the age of 62 to 72 weeks. The maximum life span of mice is more than the age within this range, but mice within this age range and even somewhat younger age have been show to express significant aging-related susceptibility to respiratory disease by influenza virus, RSV and SARS coronavirus^55–57^, declined immune responsiveness^55,58^ and a broad range of other age-related aberrations^37,38^. We choose to use mice within this age range since they would show aging-related phenomena, without the risk of losing mice from our analyses due to very old age^37,38^. Mice were kept in a 12-hour light/dark cycle under SPF and temperature-controlled conditions, and were fed *ad libitum*.

### Ethics

All animal work was approved by the ethics committee (Dierexperimentencommissie) of RIVM (Rijksintituut voor Volksgezondheid en Milieu) and conducted in accordance with the Dutch national legislation on Animal Experimentation.

### Experimental Design

Experimental procedures have been described extensively in our previous studies and were performed in accordance with national and our institutional guidelines^5,59^. In brief, young and old C57BL/6 mice were inoculated intranasally with 50 µl containing an infectious dose of 10^6^ TCID_50_ RSV type A2 or mock as control, under anaesthesia by isoflurane. At day 2 and 5 after inoculation, mice were anesthetized with Ketamine/Xylazine, bled and sacrificed to collect lungs. Lungs were perfused with PBS to eliminate blood cells prior to collection of the tissue, immersed in RNAlater RNA stabilization agent (Ambion) and subsequently stored at −80 °C until further processing. RNA was extracted from homogenized lung tissue using the RNeasy kit (Qiagen). RNA concentrations were measured using a NanoDrop spectrophotometer (Thermo Fisher Scientific). RNA quality was measured using the Bioanalyzer (Agilent Technologies). A total number of 28 RNA samples (N = 4 for uninfected groups, N = 5 for infected groups) were used for transcriptional profiling with microarrays.

### Microarray analysis

RNA amplification, labelling and hybridization to microarrays was carried out at the Microarray Department of the University of Amsterdam, The Netherlands, according to methods described previously^60^. Cy3-labeled RNA samples and a Cy5-labeled common reference (made by pooling equimolar amounts of antisense RNA aRNA from individual samples) were hybridized to NimbleGen 12 × 135 k *Mus musculus* microarrays (Roche, Germany), containing probes for 44,170 genes with 3 spots per target probe. Each microarray corresponded to labelled RNA from one individual mouse.

Briefly, 500 ng total RNA of each sample was amplified according to the Agilent QuickAmp kit manual (Agilent technologies). A common reference sample was made by pooling equimolar amounts of aRNA from individual samples. Cy3 and Cy5 monoreactive dyes (GE Healthcare) were used to label individual samples and the common reference sample, respectively. Labelling was performed with 10 ml of CyDye solution and incubated for 1 hour before the reaction was quenched by adding 5 ml 4 M hydroxylamine (Sigma-Aldrich). Purification was performed by using a clean-up kit (E.Z.N.A. MicroElute RNA Clean Up Kit, Omega Bio-Tek, Norcross, United States). The yields of amplified RNA and incorporation of CyDye were determined by using a NanoDrop spectrophotometer. Hybridization mixture was prepared by adding a dried mixture (1:1) of sample (Cy3) and common reference (Cy5) together with sample tracking control (STC, Roche NimbleGen) and hybridization cocktail (NimbleGen Arrays User’s Guide – Gene Expression Arrays Version 5.0, Roche NimbleGen). Samples were incubated for 5 min. at 95 °C and 5 min. at 42 °C. Hybridization was performed by loading a sample onto a microarray. Hybridization was performed with a NimbleGen Hybridization System 4 (Roche NimbleGen) for 20 hours at 42 °C. After washing (NimbleGen Arrays User’s Guide – Gene Expression Arrays Version 5.0), slides were scanned in an ozone-free room with a microarray scanner (Agilent DNA microarray scanner G2565CA, Agilent Technologies). Feature extraction was performed with NimbleScan v2.5 (Roche NimbleGen) resulting in a table containing individual probe signal intensities for both dyes.

Complete raw and normalized microarray data and their MIAME compliant metadata from this publication have been submitted to the GEO database (www.ncbi.nlm.nih.gov/geo) and assigned the identifier GSE111947.

### Data analysis

Quality control on raw microarray data was performed by means of Cy3-Cy5 scatter plots and by comparing signal average and distribution across slides. All slides passed quality control. Data for gene-coding probes were normalized in R (www.r-project.org), by using a four step approach^60^ consisting of: (1) natural log-transformation, (2) quantile normalization of all scans, (3) correcting the sample spot signal for its corresponding reference spot signal and (4) averaging data from replicate probe spots. This resulted in normalized data for 44170 probes, which was used for further statistical analysis in R and Microsoft Excel.

Differentially expressed genes were identified by using ANOVA to compare (a) groups of young mice (uninfected, 2 days, 5 days post infection (p.i.)); (b) groups of old mice (uninfected, 2 days, 5 days p.i.); (c) uninfected young vs old mice. Probes were considered differentially expressed if they met the following two criteria for at least one of the above comparisons: (i) a p-value < 0.001 (determined by ANOVA), which for this study corresponds to a False discovery rate (FDR) < 5% when accounting for multiple testing; and (ii) an absolute fold change > 1.5 (up- or down-regulated). If multiple probes corresponding to the same gene were significant, their data were averaged to remove redundancy in further analyses. Fold changes were calculated as the average gene expression levels of three mice per group compared to the respective control group. Variation within groups was expressed as relative standard deviation (also known as the coefficient of variation) and calculated as the square root of the average variance across the expression data per gene within each group. The complete set of all significantly regulated genes can be found in a supplemental file (Table S1) accompanying this paper.

Differences in gene expression were visualized by Principal Component Analysis (PCA) and by heatmaps. Functional annotation with over-representation analysis of differentially expressed genes was carried out by using DAVID^24^. Genes with similar functional annotation based on Gene Ontology (GO), Kyoto Encyclopedia of Genes and Genomes (KEGG), or UniProt, were combined into functional umbrella terms. Additionally, differentially expressed genes were imported in Ingenuity Pathway Analysis (Qiagen), Build version: 463341 M, Content version: 42012434 (Release Date: 2017-12-07), for prediction of upstream regulators as well as comparison with diseases and biological functions.

### Viral load

RSV was quantified in the lungs of mice by real-time quantitative PCR (RT Q-PCR) for detection of RNA copies of RSV A as described before^5^, using the 7500 Fast real-time PCR system (Thermo Fisher Scientific). All PCRs were done on equal amounts of input RNA, calculated from the total RNA concentrations measured in each sample. Expression of the housekeeping gene hypoxanthine phosphoribosyltransferase (*Hprt*) was used as an endogenous reference, and confirmed equal amounts of total RNA used as input for all different samples. Threshold cycles (C_T_) values were used for quantification of RNA copies of RSV or HPRT during runs of 40 PCR cycles. The amount of RSV RNA was quantified by calculating the C_T_ values for RSV relative to the C_T_ values measured for *Hprt* using the 2^ΔCT^ method.

### Real time Q-PCR validation

To validate the gene expression changes found by microarray analysis, we performed real time quantitative PCR (RT Q-PCR). For this purpose, ten genes were chosen that represent the various pathways affected, namely innate interferon (IFN)-mediated antiviral function (*Ifit1*, *Mx1*, *Mx2*), cytokines (*Ccl8*, *Cxcl9*, *Cxcl10*), antigen processing and presentation (*B2m*), other immune function (*Saa3*), and matrix constitution (*Col1a2*, *Sparc*). All reagents, methods and equipment were obtained from Thermo Fisher Scientific. TaqMan gene expression assay IDs used are given in Supplemental Table S3. Assays for *Hprt* (custom made^5^) and *Polr2a* (Mm00839502_m1) were included as endogenous controls. Threshold cycles were automatically derived from the amplification plots constructed of the fluorescence signals by the 7500 Fast system SDS software. The average of the *Hprt* and *Polr2a* level per sample was used to normalize the expression of the other genes. Relative quantification of the mRNA copies in the samples compared to that of the young mock-infected group was performed by the comparative threshold cycle (2−ΔΔCT) method using Microsoft Excel.

## Electronic supplementary material

Supplementary tables 1–3
