# Supplementary material for: Transcriptomics in lung tissue upon respiratory syncytial virus infection reveals aging as important modulator of immune activation and matrix maintenance
Source: Sci Rep. 2018 Nov 9;8:16653. doi: 10.1038/s41598-018-35180-2 (PMC6226529; doi:10.1038/s41598-018-35180-2)
Supplement: Supplementary file 1 — Supplementary tables 1–3 [file 41598_2018_35180_MOESM1_ESM.pdf]

SUPPLEMENTARY TABLES

Transcriptomics in lung tissue upon respiratory syncytial virus infection reveals aging as important modulator of immune activation and matrix maintenance

Jeroen L.A. Pennings, Rob Mariman, Hennie M. Hodemaekers, Sylvia S.N. Reemers, Riny Janssen, Teun Guichelaar

TABLE S1. Transcriptome of all significantly regulated genes

Symbol	YM0/YM0	YR2/YM0	YR5/YM0	OM0/YM0	OR2/YM0	OR5/YM0	OR2/OM0	OR5/OM0	RSV young signif?	RSV old signif?	age signif?	GeneID	Functional category	Synonyms	Chromosome	Gene name	type_of_gene
Isg15	1	3.49	1.50	0.85	4.78	1.40	5.66	1.66	up	up	ns	100038882	innate antiviral/IFN	G1p2|IGI15|IP17|Irfp|UCRP	4	ISG15 ubiquitin-like modifier	protein-coding
Ifi27l2a	1	3.88	1.25	0.88	5.56	1.22	6.33	1.38	up	up	ns	76933	innate antiviral/IFN	2310061N23Rik|Ifi27|Isg12|Isg12(b1)	12	interferon, alpha-inducible protein 27 like 2A	protein-coding
Irf7	1	3.74	1.50	0.98	5.63	1.49	5.74	1.52	up	up	ns	54123	innate antiviral/IFN	-	7	interferon regulatory factor 7	protein-coding
Apod	1	3.98	1.50	1.12	6.29	1.59	5.63	1.42	up	up	ns	11815	other	-	16	apolipoprotein D	protein-coding
Ifit3	1	3.43	1.74	1.02	4.47	1.41	4.40	1.39	up	up	ns	15959	innate antiviral/IFN	Ifi49|P49	19	interferon-induced protein with tetratricopeptide repeats 3	protein-coding
Ifit3b	1	3.22	1.72	1.06	4.18	1.40	3.92	1.31	up	up	ns	667370	innate antiviral/IFN	I830012O16Rik	19	interferon-induced protein with tetratricopeptide repeats 3B	protein-coding
Saa3	1	7.52	2.35	1.34	12.55	1.80	9.39	1.35	up	ns	ns	20210	immune (other)	AV098916|Saa-3|I7R3	7	serum amyloid A 3	protein-coding
Mx2	1	1.91	1.19	0.93	2.39	1.27	2.57	1.36	up	up	ns	17858	innate antiviral/IFN	AI528743|Mx-2	16	MX dynamin-like GTPase 2	protein-coding
Ddx60	1	2.02	1.33	1.01	2.47	1.16	2.43	1.14	up	up	ns	234311	unknown	9830118M07	8	DEAD (Asp-Glu-Ala-Asp) box polypeptide 60	protein-coding
Mx1	1	1.97	1.36	0.99	2.38	1.35	2.39	1.35	up	up	ns	17857	innate antiviral/IFN	AI893580|Mx|Mx-1	16	MX dynamin-like GTPase 1	protein-coding
Gm5431	1	1.96	1.39	1.06	2.42	1.35	2.28	1.27	up	up	ns	432555	gene model	EG432555	11	predicted gene 5431	protein-coding
Ifit2	1	1.96	1.29	1.03	2.50	1.29	2.42	1.25	up	up	ns	15958	innate antiviral/IFN	AV302338|Ifi54|P54	19	interferon-induced protein with tetratricopeptide repeats 2	protein-coding
Cmpk2	1	1.84	1.39	1.00	2.21	1.27	2.21	1.26	ns	up	ns	22169	other	1200004E04Rik|TDKI|Tyki	12	cytidine monophosphate (UMP-CMP) kinase 2, mitochondrial	protein-coding
Ifi44	1	2.14	1.37	0.73	2.68	1.05	3.65	1.43	up	up	ns	99899	innate antiviral/IFN	A430056A10Rik|AW261460|MTAP44|p44	3	interferon-induced protein 44	protein-coding
Oasl2	1	2.19	1.40	0.97	3.05	1.25	3.15	1.29	up	up	ns	23962	innate antiviral/IFN	M1204|Mmu-OASL|Oasl	5	2'-5' oligoadenylate synthetase-like 2	protein-coding
Lgals3bp	1	2.26	1.29	1.13	3.02	1.40	2.67	1.24	up	up	ns	19039	matrix	90K|CyCAP|MAC-2BP|Ppicap|Tango10b	11	lectin, galactoside-binding, soluble, 3 binding protein	protein-coding
Ifi204	1	2.14	1.51	1.05	3.14	1.59	2.99	1.52	ns	up	ns	15951	innate antiviral/IFN	Ifi16|p204	1	interferon activated gene 204	protein-coding
Rtp4	1	2.54	1.45	0.95	3.13	1.33	3.31	1.41	up	up	ns	67775	other	5830458K16Rik|Ifrg28	16	receptor transporter protein 4	protein-coding
Ifit1	1	2.69	1.43	1.00	3.56	1.41	3.54	1.41	up	up	ns	15957	innate antiviral/IFN	ISG56|Ifi56|P56	19	interferon-induced protein with tetratricopeptide repeats 1	protein-coding
Retnla	1	2.52	1.58	0.98	2.34	1.40	2.38	1.42	up	ns	ns	57262	other	1810019L16Rik|Fizz-1|Fizz1|HIMF|RELM-alpha|RELMa|RELMalpha|Xcp2	16	resistin like alpha	protein-coding
Hhex	1	1.84	1.90	1.61	1.87	1.84	1.17	1.14	up	ns	up	15242	other	Hex|Hex1|Hhex-rs2|Prh|Prhx	19	hematopoietically expressed homeobox	protein-coding
Gata3	1	1.80	1.68	1.79	1.96	1.92	1.09	1.07	up	ns	up	14462	immune (other)	Gata-3|jal	2	GATA binding protein 3	protein-coding
Shisa2	1	1.91	1.84	1.39	2.06	1.78	1.48	1.28	up	ns	ns	219134	other	9430059P22Rik|MAd2|Tmem46|mShisa|shisa	14	shisa family member 2	protein-coding
Clec12a	1	1.88	1.77	1.34	2.39	1.92	1.78	1.43	up	ns	ns	232413	other	CLL-1|D230024O04|KLRL1|Micl	6	C-type lectin domain family 12, member a	protein-coding
Iigp1	1	2.52	2.14	1.46	2.56	1.73	1.75	1.18	up	ns	ns	60440	innate antiviral/IFN	2900074L10Rik|AI046432|AW111922|Ifgga1|Iigp|Irga6	18	interferon inducible GTPase 1	protein-coding
Rnase6	1	1.49	1.61	1.55	1.78	1.89	1.15	1.22	up	ns	ns	78416	other	9530043P15Rik|BB119466	14	ribonuclease, RNase A family, 6	protein-coding
Ltb	1	1.53	1.56	1.48	1.78	1.98	1.20	1.34	up	ns	ns	16994	cytokine	AI662801|LTbeta|Tnfc|Tnfsf3|Tnlg1c|p33	17	lymphotoxin B	protein-coding
Cd48	1	1.52	1.78	1.33	1.87	1.90	1.40	1.43	up	ns	ns	12506	other	AI449234|AW610730|BCM1|BLAST|BLAST-1|BLAST1|Bcm-1|MEM-102|SLAMF2|Sgp-60	1	CD48 antigen	protein-coding
S100a4	1	1.57	1.47	1.28	2.10	1.88	1.65	1.48	up	up	ns	20198	other	18A2|42a|Capl|FSp1|Mts1|PeL98|metastasin|pk9a	3	S100 calcium binding protein A4	protein-coding
Lair1	1	1.52	1.54	1.37	2.08	1.85	1.52	1.35	up	ns	ns	52855	other	5133400O11Rik|BB115266|D7Bwg0421e|Lair-1	7	leukocyte-associated Ig-like receptor 1	protein-coding
Ly86	1	1.56	1.52	1.29	1.96	1.75	1.53	1.36	up	ns	ns	17084	immune (other)	MD-1|MD1|ly86_tv2	13	lymphocyte antigen 86	protein-coding
Fam26f	1	1.58	1.57	1.43	2.09	2.02	1.47	1.42	up	ns	ns	215900	other	A630077B13Rik	10	family with sequence similarity 26, member F	protein-coding
Epsti1	1	1.69	1.47	1.13	2.25	1.65	1.99	1.46	up	ns	ns	108670	unknown	2310046K10Rik|5033415K03Rik|BRESI1	14	epithelial stromal interaction 1 (breast)	protein-coding
Ctss	1	1.79	1.60	1.09	1.99	1.69	1.82	1.55	up	ns	ns	13040	immune (other)	Cats	3	cathepsin S	protein-coding
Plac8	1	1.89	1.74	1.11	2.51	1.67	2.25	1.50	ns	up	ns	231507	unknown	C15|D5Wsu111e	5	placenta-specific 8	protein-coding
Mnda	1	1.76	1.31	1.17	2.52	1.62	2.16	1.39	up	up	ns	381308	other	A730048F03|Ifi205b|ifi-205-B	1	myeloid cell nuclear differentiation antigen	protein-coding
Ccl2	1	1.80	1.14	1.41	2.53	1.56	1.79	1.11	up	ns	ns	20296	cytokine	AI323594|HC11|JE|MCAF|MCP-1|MCP1|SMC-CF|Scya2|Sigje	11	chemokine (C-C motif) ligand 2	protein-coding
H2-K1	1	1.62	1.58	1.42	2.10	1.68	1.48	1.19	up	ns	ns	14972	antigen proc&pres	H-2K|H-2K(d)|H2-K|K-f	17	histocompatibility 2, K1, K region	protein-coding
B2m	1	1.68	1.55	1.32	2.06	1.57	1.56	1.18	up	ns	ns	12010	antigen proc&pres	Ly-m11|beta2-m|beta2m	2	beta-2 microglobulin	protein-coding
H2-Q4	1	1.54	1.44	1.34	2.03	1.55	1.51	1.15	up	ns	ns	15015	antigen proc&pres	H-2Q4|H2-Gs10|Qa-4|Qa4|Qat-4|Qb-1|Qb1	17	histocompatibility 2, Q region locus 4	protein-coding
Slfn10-ps	1	1.63	1.39	1.38	2.28	1.58	1.66	1.15	up	ns	ns	237887	unknown	Slfn10|mFLJ00257	11	schlafen 10, pseudogene	pseudo
Evi2a	1	1.57	1.42	1.44	1.83	1.70	1.28	1.18	up	ns	ns	14017	other	AW491894|Evi-2|Evi2	11	ecotropic viral integration site 2a	protein-coding
Cx3cr1	1	1.58	1.51	1.40	1.78	1.59	1.27	1.14	up	ns	ns	13051	immune (other)	-	9	chemokine (C-X3-C motif) receptor 1	protein-coding
Clec2i	1	1.48	1.57	1.36	1.65	1.56	1.21	1.14	up	ns	ns	93675	other	Clr-g|Clrg|Dcl1|LCL-1|OCILrP2	6	C-type lectin domain family 2, member i	protein-coding
Sp100	1	1.68	1.34	1.21	2.09	1.41	1.73	1.17	up	up	ns	20684	other	A430075G10Rik	1	nuclear antigen Sp100	protein-coding
Ifi203	1	1.62	1.32	1.12	2.05	1.48	1.83	1.31	up	up	ns	15950	innate antiviral/IFN	-	1	interferon activated gene 203	protein-coding
H2-D1	1	1.55	1.43	1.28	1.84	1.41	1.44	1.11	up	ns	ns	14964	antigen proc&pres	H-2D|H2-D	17	histocompatibility 2, D region locus 1	protein-coding
Glipr1	1	1.17	1.37	1.62	1.78	2.01	1.10	1.24	ns	ns	up	73690	other	2410114O14Rik|RTVP-1|RTVP1|mRTVP-1	10	GLI pathogenesis-related 1 (glioma)	protein-coding
Cxcr6	1	1.25	1.41	1.84	1.96	2.13	1.06	1.15	ns	ns	up	80901	immune (other)	BB217514|BONZO|STRL33	9	chemokine (C-X-C motif) receptor 6	protein-coding
Agrp	1	1.16	1.22	1.99	2.24	2.13	1.12	1.07	ns	ns	up	11604	other	Agrt|Art	8	agouti related neuropeptide	protein-coding
Gp2	1	1.39	1.39	1.69	2.33	2.08	1.37	1.23	ns	ns	up	67133	other	2310037I18Rik|AV060639	7	glycoprotein 2 (zymogen granule membrane)	protein-coding
Cybb	1	1.44	1.62	1.61	1.98	2.22	1.23	1.38	up	ns	up	13058	immune (other)	C88302|CGD91-phox|Cgd|Cyd|Nox2|gp91-1|gp91phox	X	cytochrome b-245, beta polypeptide	protein-coding
Sp110	1	1.34	1.25	1.63	2.12	1.84	1.30	1.13	ns	ns	up	109032	immune (other)	5031415C07Rik|52kDa|5830484A20Rik|ENSMUSG00000075603|Ifi75|Ipr1	1	Sp110 nuclear body protein	protein-coding
Cd177	1	1.38	1.29	1.51	2.12	1.87	1.41	1.24	ns	ns	up	68891	other	1190003K14Rik|Pdp3	7	CD177 antigen	protein-coding
C4b	1	1.52	1.20	1.59	2.31	1.73	1.45	1.09	up	ns	up	12268	immune (other)	C4|Ss	17	complement component 4B (Chido blood group)	protein-coding
Ms4a7	1	1.70	2.39	1.72	2.89	3.20	1.68	1.86	ns	ns	up	109225	other	9130422I10Rik|A430103C15Rik|CD20l4|CFFMA	19	membrane-spanning 4-domains, subfamily A, member 7	protein-coding
Aif1	1	1.88	1.67	1.53	2.95	2.70	1.93	1.76	up	ns	ns	11629	other	AI607846|AIF-1|D17H6S50E|G1|Iba1	17	allograft inflammatory factor 1	protein-coding
Zbp1	1	1.96	1.39	1.29	2.86	1.68	2.22	1.30	up	up	ns	58203	innate antiviral/IFN	2010010H03Rik|Dai|Dlm1|mZaDLM	2	Z-DNA binding protein 1	protein-coding
Cxcl10	1	2.03	1.37	1.38	3.06	1.96	2.21	1.42	up	ns	ns	15945	cytokine	C7|CRG-2|INP10|IP-10|IP10|Ifi10|Scyb10|gIP-10|mob-1	5	chemokine (C-X-C motif) ligand 10	protein-coding
Ccl7	1	1.95	1.25	1.61	2.87	1.90	1.78	1.18	up	ns	ns	20306	cytokine	MCP-3|Scya7|fic|marc|mcp3	11	chemokine (C-C motif) ligand 7	protein-coding
Cfb	1	2.15	1.36	1.56	3.67	1.81	2.36	1.16	up	ns	ns	14962	immune (other)	AI195813|AI255840|B|Bf|C2|Fb|H2-Bf	17	complement factor B	protein-coding
Rsad2	1	2.19	1.46	1.39	3.03	1.39	2.18	1.00	up	up	ns	58185	innate antiviral/IFN	2510004L01Rik|Vig1|cig5	12	radical S-adenosyl methionine domain containing 2	protein-coding
Timp1	1	1.72	1.06	0.77	1.86	0.85	2.41	1.10	up	up	ns	21857	matrix	Clgi|EPA|TIMP-1|TPA-S1|Timp	X	tissue inhibitor of metalloproteinase 1	protein-coding
Pydc4	1	1.29	1.16	0.82	1.41	0.83	1.73	1.01	ns	up	ns	623121	unknown	E030037K03Rik|E430029J22Rik	1	pyrin domain containing 4	protein-coding
Irf9	1	1.46	1.16	0.95	1.58	1.04	1.66	1.09	up	up	ns	16391	innate antiviral/IFN	Irf-9|Isgf3g|p48	14	interferon regulatory factor 9	protein-coding
Herc6	1	1.43	1.12	0.87	1.61	1.08	1.86	1.25	ns	up	ns	67138	unknown	1700121D12Rik|2510038N07Rik|4930427L17Rik|AI451296|CEB1|Herc5	6	hect domain and RLD 6	protein-coding
Eif2ak2	1	1.46	1.04	0.96	1.70	1.04	1.78	1.08	ns	up	ns	19106	innate antiviral/IFN	2310047A08Rik|4732414G15Rik|AI467567|AI747578|Pkr|Prkr|Tik	17	eukaryotic translation initiation factor 2-alpha kinase 2	protein-coding
Trim12a	1	1.36	1.24	1.00	1.54	1.13	1.54	1.13	ns	up	ns	76681	unknown	2310043C01Rik|Trim12	7	tripartite motif-containing 12A	protein-coding
Dhx58	1	1.36	1.18	1.01	1.61	1.10	1.60	1.09	ns	up	ns	80861	immune (other)	B430001I08Rik|D11Lgp2e|LPG2|Lgp2|RLR-3	11	DEXH (Asp-Glu-X-His) box polypeptide 58	protein-coding
Rnf213	1	1.67	1.26	0.92	1.87	1.12	2.04	1.22	up	up	ns	672511	unknown	6030403J01|D11Ertd759e|mKIAA1554	11	ring finger protein 213	protein-coding
Adar	1	1.62	1.21	0.99	1.69	1.11	1.71	1.13	up	up	ns	56417	other	AV242451|Adar1|Adar1p110|Adar1p150|mZaADAR	3	adenosine deaminase, RNA-specific	protein-coding
Oasl1	1	1.52	1.15	1.00	1.91	1.13	1.91	1.13	up	up	ns	231655	innate antiviral/IFN	7530414C13Rik|oasl9	5	2'-5' oligoadenylate synthetase-like 1	protein-coding
Ifih1	1	1.51	1.14	0.95	1.79	1.14	1.87	1.19	up	up	ns	71586	innate antiviral/IFN	9130009C22Rik|Helicard|Hlcd|MDA5|RLR-2	2	interferon induced with helicase C domain 1	protein-coding
Xaf1	1	1.76	1.14	0.91	2.06	1.08	2.28	1.19	up	up	ns	327959	other	Fbox39	11	XIAP associated factor 1	protein-coding
Phf11d	1	1.73	1.18	0.93	2.20	1.16	2.37	1.25	up	up	ns	219132	unknown	D14Ertd668e|Phf11|Phf11l	14	PHD finger protein 11D	protein-coding
Ifitm3	1	1.68	1.14	0.99	2.01	1.12	2.02	1.13	up	up	ns	66141	innate antiviral/IFN	1110004C05Rik|Cd225|Cdw217|DSPA2b|Fgls|IP15|mil-1	7	interferon induced transmembrane protein 3	protein-coding
Bst2	1	1.68	1.17	0.96	2.05	1.10	2.14	1.15	up	up	ns	69550	other	2310015I10Rik|Bst-2|C87040|CD317|DAMP-1|GREG	8	bone marrow stromal cell antigen 2	protein-coding
Oas1g	1	1.33	1.12	1.03	1.68	1.16	1.63	1.12	ns	up	ns	23960	innate antiviral/IFN	AI449562|L2|Mmu-L|Mmu-L2|Oas1b|Oias-1|Oias1	5	2'-5' oligoadenylate synthetase 1G	protein-coding
Ly6e	1	1.44	1.10	1.11	1.74	1.20	1.57	1.08	ns	up	ns	17069	other	Ly67|RIG-E|Sca-2|TSA-1|Tsa1	15	lymphocyte antigen 6 complex, locus E	protein-coding
Trex1	1	1.41	1.17	1.08	1.69	1.34	1.56	1.24	ns	up	ns	22040	other	AU041952	9	three prime repair exonuclease 1	protein-coding
C1rb	1	1.42	1.26	1.09	1.66	1.28	1.52	1.17	ns	up	ns	667277	immune (other)	Gm8551|mC1rB	6	complement component 1, r subcomponent B	protein-coding
Parp9	1	1.45	1.19	1.04	1.69	1.22	1.62	1.17	ns	up	ns	80285	other	ARTD9|AW214463|BC003281|Bagl|Bal|PARP-9	16	poly (ADP-ribose) polymerase family, member 9	protein-coding
Ms4a6d	1	1.52	1.23	0.97	1.67	1.42	1.72	1.46	up	ns	ns	68774	other	1110058E16Rik|AI447446|Ms4a11	19	membrane-spanning 4-domains, subfamily A, member 6D	protein-coding
Fcgr1	1	1.61	1.29	1.01	1.73	1.27	1.70	1.26	up	ns	ns	14129	antigen proc&pres	AI323638|AV092959|CD64|FcgammaRI|IGGHAFC	3	Fc receptor, IgG, high affinity I	protein-coding
Trim30d	1	1.51	1.13	1.13	1.89	1.34	1.68	1.19	up	up	ns	209387	unknown	AI451617|TRIM30-3|Trim79	7	tripartite motif-containing 30D	protein-coding
Oas1a	1	1.49	1.16	1.07	2.09	1.27	1.96	1.19	ns	up	ns	246730	innate antiviral/IFN	L3	5	2'-5' oligoadenylate synthetase 1A	protein-coding
Trim30a	1	1.51	1.31	1.08	1.96	1.31	1.82	1.21	up	up	ns	20128	unknown	Rpt-1|Rpt1|Trim30	7	tripartite motif-containing 30A	protein-coding
Phf11a	1	1.43	1.22	1.07	1.93	1.29	1.80	1.21	ns	up	ns	219131	unknown	4933417L10Rik|Phf11|Phf11l	14	PHD finger protein 11A	protein-coding
Parp14	1	1.62	1.22	1.16	1.77	1.19	1.53	1.03	up	ns	ns	547253	other	1600029O10Rik|ARTD8|BC021340|CoaSt6|mKIAA1268	16	poly (ADP-ribose) polymerase family, member 14	protein-coding
Apol9b	1	1.27	1.05	1.19	1.88	1.32	1.58	1.11	ns	up	ns	71898	other	2310016F22Rik	15	apolipoprotein L 9b	protein-coding
Apol9a	1	1.33	1.12	1.21	2.01	1.36	1.67	1.12	ns	up	ns	223672	other	-	15	apolipoprotein L 9a	protein-coding
Oas2	1	1.42	1.18	1.27	1.99	1.42	1.56	1.12	ns	up	ns	246728	innate antiviral/IFN	Oasl11	5	2'-5' oligoadenylate synthetase 2	protein-coding
Fos	1	2.68	0.80	1.02	1.36	1.44	1.33	1.41	up	ns	ns	14281	other	D12Rfj1|c-fos|cFos	12	FBJ osteosarcoma oncogene	protein-coding
Cyr61	1	2.47	0.78	1.20	1.31	1.13	1.09	0.94	up	ns	ns	16007	other	AI325051|CCN1|Igfbp10	3	cysteine rich protein 61	protein-coding
Flnb	1	0.98	1.05	0.90	1.42	1.22	1.57	1.35	ns	up	ns	286940	other	AL024016|Fln-b	14	filamin, beta	protein-coding
Fam46a	1	1.20	1.23	0.89	1.40	1.27	1.57	1.43	ns	up	ns	212943	unknown	D930050G01Rik	9	family with sequence similarity 46, member A	protein-coding
Wisp2	1	1.26	1.52	0.93	1.19	1.09	1.28	1.16	up	ns	ns	22403	other	Ccn5|Crgr4|Ctgfl|Rcop1	2	WNT1 inducible signaling pathway protein 2	protein-coding
Tmtc2	1	1.41	1.56	0.59	1.25	1.23	2.11	2.07	up	up	ns	278279	other	8430438D04Rik|D330034A10Rik	10	transmembrane and tetratricopeptide repeat containing 2	protein-coding
Plcb1	1	0.92	1.03	0.59	1.25	0.89	2.12	1.51	ns	up	dn	18795	other	3110043I21Rik|AI132408|Plcb|mKIAA0581	2	phospholipase C, beta 1	protein-coding
St8sia4	1	1.45	1.54	1.09	1.41	1.21	1.29	1.11	up	ns	ns	20452	other	PST|PST-1|SIAT8-D|ST8SiaIV|Siat8d	1	ST8 alpha-N-acetyl-neuraminide alpha-2,8-sialyltransferase 4	protein-coding
Gpsm2	1	1.52	1.36	1.06	1.26	1.13	1.18	1.06	up	ns	ns	76123	other	6230410J09Rik|LGN|Pins	3	G-protein signalling modulator 2 (AGS3-like, C. elegans)	protein-coding
Mylip	1	1.53	1.43	1.16	1.19	1.27	1.03	1.10	up	ns	ns	218203	other	9430057C20Rik|Mir	13	myosin regulatory light chain interacting protein	protein-coding
Casc4	1	1.64	1.46	1.25	1.22	1.21	0.98	0.97	up	ns	ns	319996	other	D130060C09Rik|VGFG2573	2	cancer susceptibility candidate 4	protein-coding
Zeb2	1	1.52	1.36	1.23	1.31	1.28	1.06	1.04	up	ns	ns	24136	other	9130203F04Rik|D130016B08Rik|SIP1|Zfhx1b|Zfx1b|Zfxh1b	2	zinc finger E-box binding homeobox 2	protein-coding
Prr5l	1	1.53	1.46	1.29	1.31	1.14	1.01	0.88	up	ns	ns	72446	other	2600010E01Rik|4833411O04Rik|Protor-2|Protor2	2	proline rich 5 like	protein-coding
Rgs2	1	1.86	1.25	1.26	1.15	1.09	0.91	0.86	up	ns	ns	19735	other	GOS8	1	regulator of G-protein signaling 2	protein-coding
Stk4	1	1.48	1.41	1.53	1.43	1.38	0.93	0.90	ns	ns	up	58231	other	AI447339|AU020804|Kas-2|Mst1|Ysk3	2	serine/threonine kinase 4	protein-coding
Rpl36a-ps2	1	1.40	1.49	1.66	1.39	1.54	0.84	0.93	ns	ns	up	100040331	unknown	Gm2718	3	ribosomal protein L36A, pseudogene 2	pseudo
Id2	1	1.51	1.45	1.34	1.30	1.42	0.97	1.06	up	ns	ns	15902	other	AI255428|C78922|Idb2|bHLHb26	12	inhibitor of DNA binding 2	protein-coding
Bmp7	1	1.51	1.27	1.42	1.29	1.39	0.91	0.98	up	ns	ns	12162	cytokine	OP1	2	bone morphogenetic protein 7	protein-coding
Sox4	1	1.79	1.61	1.30	1.35	1.27	1.04	0.98	up	ns	ns	20677	other	AA682046|Sox-4	13	SRY (sex determining region Y)-box 4	protein-coding
Meox2	1	1.85	1.65	1.30	1.63	1.46	1.26	1.13	up	ns	ns	17286	other	AI528662|Gax|Mox-2|Mox2	12	mesenchyme homeobox 2	protein-coding
Sirpb1a	1	1.64	1.59	1.15	1.68	1.51	1.45	1.31	up	ns	ns	320832	other	9930027N05Rik|SIRP-beta|Sirpb|Sirpb1	3	signal-regulatory protein beta 1A	protein-coding
Msr1	1	1.58	1.52	1.10	1.71	1.51	1.56	1.37	up	ns	ns	20288	other	MRS-A|MSR|MSR-A|SR-AI|SR-AII|Scara1|Scvr	8	macrophage scavenger receptor 1	protein-coding
Clec7a	1	1.66	1.60	1.05	1.56	1.42	1.48	1.35	up	ns	ns	56644	immune (other)	BGR|Clecsf12|beta-GR	6	C-type lectin domain family 7, member a	protein-coding
Bbox1	1	0.87	0.83	2.27	1.97	2.06	0.87	0.91	ns	ns	up	170442	other	AI874739	2	butyrobetaine (gamma), 2-oxoglutarate dioxygenase 1 (gamma-butyrobetaine hydroxylase)	protein-coding
1700016D02Rik	1	0.85	0.82	2.40	1.85	1.77	0.77	0.74	ns	ns	up	75535	unknown	-	14	RIKEN cDNA 1700016D02 gene	protein-coding
Gm8042	1	0.89	0.83	2.17	1.67	1.57	0.77	0.72	ns	ns	up	666318	gene model	_	_	_	discontinued
Gm7999	1	0.85	0.79	2.18	1.71	1.56	0.78	0.72	ns	ns	up	666239	gene model	_	_	_	discontinued
Apol11b	1	1.09	1.29	2.88	1.94	1.62	0.67	0.56	ns	ns	up	328563	other	A330102K04Rik	15	apolipoprotein L 11b	protein-coding
Calml4	1	0.90	0.99	1.81	1.60	1.68	0.88	0.93	ns	ns	up	75600	other	2010002G05Rik	9	calmodulin-like 4	protein-coding
Azgp1	1	0.97	0.95	1.78	1.57	1.68	0.88	0.94	ns	ns	up	12007	antigen proc&pres	Zag	5	alpha-2-glycoprotein 1, zinc	protein-coding
Gm7995	1	1.00	0.96	1.95	1.63	1.56	0.83	0.80	ns	ns	up	666233	gene model	100041950|Gm3586	14	predicted gene 7995	protein-coding
Gm3522	1	0.92	0.91	1.86	1.49	1.50	0.80	0.81	ns	ns	up	100041810	gene model	_	_	_	discontinued
Gm3161	1	0.89	0.86	1.93	1.45	1.48	0.75	0.76	ns	ns	up	100041143	gene model	-	14	predicted gene 3161	unknown
Nxph1	1	0.94	0.88	1.97	1.50	1.60	0.76	0.81	ns	ns	up	18231	other	C130005L03Rik	6	neurexophilin 1	protein-coding
Got1l1	1	0.90	0.98	1.76	1.45	1.47	0.83	0.84	ns	ns	up	76615	other	1700083M11Rik	8	glutamic-oxaloacetic transaminase 1-like 1	protein-coding
Fam183b	1	0.94	0.99	1.66	1.48	1.50	0.89	0.90	ns	ns	up	75429	unknown	3100002J23Rik|Theg6	11	family with sequence similarity 183, member B	protein-coding
Vwc2	1	0.97	0.93	1.68	1.54	1.54	0.92	0.92	ns	ns	up	319922	matrix	A930041G11Rik|G11|PSST739|UNQ739|cradin	11	von Willebrand factor C domain containing 2	protein-coding
Sult2a1	1	0.98	1.02	1.78	1.46	1.40	0.82	0.79	ns	ns	up	20859	other	ST2A1|Std|Sth1|mSTa1	7	sulfotransferase family 2A, dehydroepiandrosterone (DHEA)-preferring, member 1	protein-coding
Hmgcs2	1	0.88	1.00	1.59	1.25	1.67	0.79	1.05	ns	ns	up	15360	other	1300002P16|mHS	3	3-hydroxy-3-methylglutaryl-Coenzyme A synthase 2	protein-coding
A930001A20Rik	1	0.87	0.92	1.74	1.36	1.58	0.79	0.91	ns	ns	up	77127	unknown	-	3	RIKEN cDNA A930001A20 gene	ncRNA
Spp1	1	0.94	1.00	1.60	1.30	1.95	0.81	1.22	ns	ns	up	20750	cytokine	2AR|Apl-1|BNSP|BSPI|Bsp|ETA-1|Eta|OP|Opn|Opnl|Ric|Spp-1	5	secreted phosphoprotein 1	protein-coding
Ppp2r2c	1	1.05	1.06	1.74	1.38	1.64	0.80	0.95	ns	ns	up	269643	other	6330548O06Rik|IMYPNO|IMYPNO1|PR52	5	protein phosphatase 2, regulatory subunit B, gamma	protein-coding
Grip2	1	1.06	1.03	1.61	1.36	1.47	0.85	0.91	ns	ns	up	243547	other	-	6	glutamate receptor interacting protein 2	protein-coding
Tmem232	1	1.01	1.03	1.59	1.54	1.62	0.97	1.01	ns	ns	up	381107	unknown	E130009J12Rik|Gm940|Tes13-L|Tes13-S	17	transmembrane protein 232	protein-coding
Esrrg	1	1.03	1.03	1.66	1.50	1.57	0.90	0.94	ns	ns	up	26381	other	ERR3|Errg|NR3B3|mKIAA0832	1	estrogen-related receptor gamma	protein-coding
Fsip1	1	1.10	1.06	1.56	1.53	1.46	0.98	0.94	ns	ns	up	71313	other	1700012M13Rik|4933432K11Rik	2	fibrous sheath-interacting protein 1	protein-coding
Ccdc153	1	1.07	1.06	1.56	1.49	1.50	0.95	0.96	ns	ns	up	270150	unknown	-	9	coiled-coil domain containing 153	protein-coding
Vpreb3	1	1.11	1.08	1.55	1.52	1.56	0.98	1.00	ns	ns	up	22364	unknown	8HS-20|AI528709|Vpreb-3	10	pre-B lymphocyte gene 3	protein-coding
Ccdc81	1	1.05	1.01	1.52	1.58	1.45	1.04	0.96	ns	ns	up	70884	unknown	4921513D09Rik	7	coiled-coil domain containing 81	protein-coding
Edn1	1	1.21	1.11	1.92	1.31	1.65	0.68	0.86	ns	ns	up	13614	other	ET-1|PPET1|preproET	13	endothelin 1	protein-coding
Tmem212	1	1.04	1.05	1.65	1.60	1.58	0.97	0.96	ns	ns	up	208613	other	E030011K20Rik|Gm564	3	transmembrane protein 212	protein-coding
Mst1	1	1.07	1.09	1.61	1.63	1.56	1.01	0.97	ns	ns	up	15235	other	D3F15S2h|D9H3F15S2|DNF15S2h|Hgfl	9	macrophage stimulating 1 (hepatocyte growth factor-like)	protein-coding
Procr	1	1.04	1.09	1.62	1.75	1.62	1.08	1.00	ns	ns	up	19124	immune (other)	AI325044|Ccca|Ccd41|Epcr	2	protein C receptor, endothelial	protein-coding
Gm6329	1	1.09	1.06	1.51	1.60	1.59	1.06	1.05	ns	ns	up	622523	gene model	EG622523	8	predicted gene 6329	ncRNA
Dkkl1	1	1.06	1.03	1.55	1.63	1.69	1.06	1.10	ns	ns	up	50722	other	AI414495|SGY|SGY-1|SGY1|Soggy|mSgy	7	dickkopf-like 1	protein-coding
Pcp4l1	1	1.08	0.98	1.60	1.64	1.58	1.03	0.99	ns	ns	up	66425	unknown	1500036F01Rik	1	Purkinje cell protein 4-like 1	protein-coding
Dmbt1	1	1.08	1.01	1.57	1.79	1.58	1.14	1.01	ns	ns	up	12945	matrix	CRP|CRP-[a]|CRP-[b]|Crpd|DBMT1|gp300|p80	7	deleted in malignant brain tumors 1	protein-coding
Tnfrsf17	1	1.08	1.10	1.60	1.55	1.82	0.97	1.14	ns	ns	up	21935	immune (other)	BCM|BCMA|Tnfrsf13|Tnfrsf13a	16	tumor necrosis factor receptor superfamily, member 17	protein-coding
Gm4242	1	1.01	1.10	1.61	1.66	2.05	1.03	1.27	ns	ns	up	100043120	gene model	-	19	predicted gene 4242	other
BC048546	1	1.11	0.97	1.89	1.63	1.89	0.86	1.00	ns	ns	up	232400	other	Ovos	6	cDNA sequence BC048546	protein-coding
Pisd-ps3	1	1.14	1.17	1.73	1.75	1.75	1.01	1.01	ns	ns	up	66776	unknown	4933439C20Rik	Y	phosphatidylserine decarboxylase, pseudogene 3	pseudo
Pisd	1	1.16	1.16	1.72	1.75	1.69	1.02	0.98	ns	ns	up	320951	other	9030221M09Rik	5	phosphatidylserine decarboxylase	protein-coding
Ankrd37	1	1.14	1.18	1.68	1.69	1.87	1.01	1.11	ns	ns	up	654824	unknown	-	8	ankyrin repeat domain 37	protein-coding
Chst9	1	0.99	1.10	2.04	2.01	1.96	0.99	0.96	ns	ns	up	71367	other	5430438D01Rik	18	carbohydrate (N-acetylgalactosamine 4-0) sulfotransferase 9	protein-coding
Cdhr1	1	1.00	1.07	1.81	1.87	1.99	1.03	1.10	ns	ns	up	170677	other	Pcdh21|Prcad|mKIAA1775	14	cadherin-related family member 1	protein-coding
Rsph1	1	0.94	0.95	1.54	1.28	1.29	0.83	0.84	ns	ns	up	22092	other	MCA|Tsga2	17	radial spoke head 1 homolog (Chlamydomonas)	protein-coding
Dnah3	1	0.97	0.98	1.52	1.26	1.27	0.83	0.84	ns	ns	up	381917	unknown	BC051401|Dnahc3	7	dynein, axonemal, heavy chain 3	protein-coding
Lrrc34	1	0.91	1.02	1.52	1.28	1.31	0.84	0.87	ns	ns	up	71827	unknown	1700007J06Rik|SPATA34	3	leucine rich repeat containing 34	protein-coding
Kndc1	1	0.91	0.99	1.61	1.40	1.38	0.87	0.86	ns	ns	up	76484	other	2410012C07Rik|B830014K08Rik|VKIND|very-kind	7	kinase non-catalytic C-lobe domain (KIND) containing 1	protein-coding
Anxa8	1	0.93	1.01	1.62	1.35	1.37	0.83	0.85	ns	ns	up	11752	other	AI466995|Anx8	14	annexin A8	protein-coding
Dnah7b	1	0.88	1.00	1.54	1.35	1.34	0.88	0.87	ns	ns	up	227058	unknown	Dnahc7b|Gm107	1	dynein, axonemal, heavy chain 7B	protein-coding
1700007K13Rik	1	0.91	0.98	1.57	1.36	1.33	0.86	0.85	ns	ns	up	69327	unknown	Rbest47	2	RIKEN cDNA 1700007K13 gene	protein-coding
Ubxn10	1	0.95	1.05	1.52	1.34	1.41	0.88	0.92	ns	ns	up	212190	unknown	5730509E04Rik|A830047D02|Ubxd3	4	UBX domain protein 10	protein-coding
1700001C02Rik	1	0.94	1.05	1.54	1.30	1.40	0.85	0.91	ns	ns	up	75434	unknown	1700047H18Rik	5	RIKEN cDNA 1700001C02 gene	protein-coding
Efcab1	1	0.92	1.05	1.50	1.34	1.46	0.89	0.97	ns	ns	up	66793	other	4930449A16Rik|5430404L10Rik	16	EF hand calcium binding domain 1	protein-coding
Dynlrb2	1	1.01	1.07	1.51	1.32	1.38	0.88	0.91	ns	ns	up	75465	other	1700009A04Rik|DNLC2B|Dncl2b	8	dynein light chain roadblock-type 2	protein-coding
Gm10419	1	0.97	0.98	1.52	1.35	1.37	0.89	0.90	ns	ns	up	100503802	gene model	6330405H19|ENSMUSG00000072769	5	predicted gene 10419	ncRNA
1110017D15Rik	1	0.95	0.93	1.52	1.35	1.39	0.89	0.91	ns	ns	up	73721	other	Cbe1|Smrp1	4	RIKEN cDNA 1110017D15 gene	protein-coding
Arhgdig	1	0.98	0.91	1.50	1.40	1.33	0.93	0.88	ns	ns	up	14570	other	Gdi5|RIP2|Rho-GDI-3|Rho-GDI2	17	Rho GDP dissociation inhibitor (GDI) gamma	protein-coding
Dmrt2	1	1.04	0.91	1.51	1.47	1.38	0.97	0.92	ns	ns	up	226049	other	Terra	19	doublesex and mab-3 related transcription factor 2	protein-coding
Cfap58	1	0.91	0.91	1.55	1.38	1.37	0.89	0.88	ns	ns	up	381229	unknown	Ccdc147|Gm969	19	cilia and flagella associated protein 58	protein-coding
Cfap161	1	0.88	0.91	1.59	1.37	1.42	0.86	0.89	ns	ns	up	75556	unknown	1700026D08Rik|AW047638|AW551802	7	cilia and flagella associated protein 161	protein-coding
Gm8090	1	0.91	0.84	1.55	1.35	1.32	0.88	0.85	ns	ns	up	666415	gene model	_	_	_	discontinued
Dnah7c	1	0.93	1.02	1.61	1.43	1.43	0.89	0.89	ns	ns	up	100101919	unknown	Dnahc7c	1	dynein, axonemal, heavy chain 7C	protein-coding
Cfap206	1	0.97	0.99	1.60	1.40	1.44	0.87	0.90	ns	ns	up	69329	unknown	1700003M02Rik|AU024269	4	cilia and flagella associated protein 206	protein-coding
Nek5	1	0.99	1.02	1.57	1.42	1.44	0.90	0.92	ns	ns	up	330721	other	6530411J22|BB049667	8	NIMA (never in mitosis gene a)-related expressed kinase 5	protein-coding
Efcab10	1	0.97	1.03	1.56	1.42	1.47	0.91	0.94	ns	ns	up	75040	unknown	4930504H06Rik	12	EF-hand calcium binding domain 10	protein-coding
1700016K19Rik	1	0.98	1.02	1.53	1.43	1.44	0.94	0.94	ns	ns	up	74230	unknown	Liat1	11	RIKEN cDNA 1700016K19 gene	protein-coding
Kif1a	1	1.01	1.02	1.54	1.45	1.48	0.94	0.96	ns	ns	up	16560	other	ATSV|C630002N23Rik|Gm1626|Kns1	1	kinesin family member 1A	protein-coding
Tekt1	1	0.95	0.96	1.52	1.41	1.48	0.93	0.97	ns	ns	up	21689	other	MT14	11	tektin 1	protein-coding
Mns1	1	0.94	0.99	1.52	1.39	1.47	0.91	0.97	ns	ns	up	17427	other	AW546487	9	meiosis-specific nuclear structural protein 1	protein-coding
Ccdc113	1	0.95	1.01	1.50	1.43	1.51	0.95	1.00	ns	ns	up	244608	unknown	4933409I22|BC060957	8	coiled-coil domain containing 113	protein-coding
Smpx	1	0.87	0.73	1.66	1.31	1.23	0.79	0.74	ns	ns	up	66106	unknown	1010001C09Rik|Csl	X	small muscle protein, X-linked	protein-coding
Scgb1c1	1	0.84	0.81	1.52	1.15	1.10	0.76	0.72	ns	ns	up	338417	other	Ryd5	7	secretoglobin, family 1C, member 1	protein-coding
Dnah6	1	0.79	0.95	1.53	1.09	1.32	0.71	0.86	ns	ns	up	330355	unknown	9830168K20|A730004I20Rik|Dnahc6|mKIAA1697|mdhc6	6	dynein, axonemal, heavy chain 6	protein-coding
Phactr1	1	1.32	1.26	1.54	1.26	1.12	0.82	0.73	ns	ns	up	218194	other	9630030F18Rik|Rpel1	13	phosphatase and actin regulator 1	protein-coding
Osbpl3	1	1.16	1.23	1.59	1.36	1.15	0.86	0.72	ns	ns	up	71720	other	1200014M06Rik|6720421I08Rik|A530055M08|ORP3|OSBP3	6	oxysterol binding protein-like 3	protein-coding

Prickle1	1	1.42	1.31	1.56	1.06	1.08	0.68	0.70	ns	ns	up	106042	other	1110058P22Rik|AW215793|Prickle|b2b019Clo|mpk1		15	prickle planar cell polarity protein 1	protein-coding
Plek	1	1.09	1.09	1.71	1.38	1.24	0.81	0.73	ns	ns	up	56193	other	2010300B13Rik		11	pleckstrin	protein-coding
Gm4621	1	1.18	1.05	1.65	1.44	1.23	0.87	0.74	ns	ns	up	100043745	gene model	_	_		_	discontinued
Htr2b	1	1.07	1.19	1.56	1.39	1.36	0.89	0.87	ns	ns	up	15559	other	5-HT2B|AJ012488|AV377389		1	5-hydroxytryptamine (serotonin) receptor 2B	protein-coding
Cxcl9	1	2.13	1.88	3.02	4.39	5.34	1.45	1.77	up	ns	up	17329	cytokine	BB139920|CMK|Mig|MuMIG|Scyb9|crg-10		5	chemokine (C-X-C motif) ligand 9	protein-coding
Ccl8	1	2.35	1.72	3.24	5.43	6.24	1.68	1.92	up	ns	ns	20307	cytokine	1810063B20Rik|AB023418|HC14|MCP-2|Mcp2|Scya8		11	chemokine (C-C motif) ligand 8	protein-coding
Gm7582	1	1.30	1.22	4.05	6.29	4.63	1.55	1.14	ns	ns	up	665317	gene model	EG665317		1	component of Sp100-rs pseudogene	pseudo
Gm2635	1	1.44	1.30	3.99	7.07	5.00	1.77	1.25	ns	ns	up	100040158	gene model	-		1	predicted pseudogene 2635	protein-coding
Csprs	1	1.35	1.29	4.72	7.12	5.36	1.51	1.14	ns	ns	up	114564	unknown	D1Lub1|HSR		1	component of Sp100-rs	protein-coding
Igkv6-23	1	1.06	1.25	3.97	4.39	6.14	1.11	1.55	ns	ns	up	637227	immunoglobulin	19-23|EG637227|Gm7202|IgK		6	immunoglobulin kappa variable 6-23	other
Igkv4-53	1	1.04	1.19	4.06	3.85	4.98	0.95	1.23	ns	ns	up	546213	immunoglobulin	Gm1420|Gm1526|IgVkkh4|kh4		6	immunoglobulin kappa variable 4-53	other
Igkv8-30	1	1.10	1.09	3.04	3.31	4.79	1.09	1.57	ns	ns	up	384419	immunoglobulin	ENSMUSG00000073025|ENSMUSG00000076577|Gm10883		6	immunoglobulin kappa chain variable 8-30	other
Speer4e	1	0.97	1.01	4.20	3.92	4.20	0.93	1.00	ns	ns	up	624245	unknown	-		5	spermatogenesis associated glutamate (E)-rich protein 4e	protein-coding
Gm9758	1	1.07	1.06	4.22	4.04	3.95	0.96	0.94	ns	ns	up	381714	gene model	ENSMUSG00000033219		5	predicted gene 9758	protein-coding
Igk	1	0.91	1.15	5.53	4.77	6.27	0.86	1.13	ns	ns	up	243469	immunoglobulin	kappa		6	immunoglobulin kappa chain complex	protein-coding
Igkv4-59	1	0.99	1.30	11.13	9.32	10.31	0.84	0.93	ns	ns	up	667683	immunoglobulin	EG667683|Gm8760|kk4		6	immunoglobulin kappa variable 4-59	other
Igkv3-4	1	1.07	1.33	11.55	9.04	11.39	0.78	0.99	ns	ns	up	626347	immunoglobulin	Igk-V21-4		6	immunoglobulin kappa variable 3-4	other
Jchain	1	0.86	1.37	11.85	10.40	13.27	0.88	1.12	ns	ns	up	16069	immunoglobulin	9530090F24Rik|AI323815|Igj|Jch		5	immunoglobulin joining chain	protein-coding
C130026I21Rik	1	1.29	1.13	2.50	2.88	2.90	1.15	1.16	ns	ns	up	620078	other	4930565N07Rik|OTTMUSG00000029174		1	RIKEN cDNA C130026I21 gene	protein-coding
A530032D15Rik	1	1.34	1.24	2.31	2.82	2.88	1.22	1.25	ns	ns	up	381287	unknown	Gm1888		1	RIKEN cDNA A530032D15Rik gene	protein-coding
Igh-VJ558	1	1.04	1.32	2.46	2.85	3.13	1.16	1.27	ns	ns	up	16061	immunoglobulin	AI893585		12	immunoglobulin heavy chain (J558 family)	other
LOC664787	1	1.36	1.19	2.98	3.88	3.47	1.30	1.16	ns	ns	up	664787	unknown	-		1	Sp110 nuclear body protein pseudogene	pseudo
Gm7592	1	1.20	1.17	2.93	3.95	3.17	1.35	1.08	ns	ns	up	665338	gene model	EG665338		1	predicted gene 7592	protein-coding
Gm15753	1	1.18	1.09	2.78	3.26	3.09	1.18	1.11	ns	ns	up	624083	gene model	OTTMUSG00000025953		5	predicted gene 15753	protein-coding
Gm17019	1	1.05	1.10	3.20	2.96	3.21	0.92	1.00	ns	ns	up	66773	gene model	-		5	predicted gene 17019	protein-coding
Speer4b	1	1.05	1.14	3.11	2.93	3.19	0.94	1.03	ns	ns	up	73526	unknown	1700081O22Rik|SPEER-4B		5	spermatogenesis associated glutamate (E)-rich protein 4B	protein-coding
Igkc	1	0.98	1.10	3.11	2.98	3.21	0.96	1.03	ns	ns	up	16071	immunoglobulin	Igk-C		6	immunoglobulin kappa constant	other
Ighg	1	0.84	0.98	2.85	3.10	3.51	1.09	1.23	ns	ns	up	380794	immunoglobulin	AU044919|IGHG2A		12	Immunoglobulin heavy chain (gamma polypeptide)	protein-coding
Ubd	1	1.02	1.12	2.09	2.08	2.71	1.00	1.30	ns	ns	up	24108	other	FAT10		17	ubiquitin D	protein-coding
Gm5862	1	0.93	1.02	2.30	2.30	2.47	1.00	1.07	ns	ns	up	545739	gene model	EG545739		5	predicted gene 5862	protein-coding
Speer4cos	1	1.04	1.10	2.45	2.42	2.55	0.99	1.04	ns	ns	up	75858	unknown	4930568L21Rik|SPEER-7|Speer7-ps|Speer7-ps1		5	spermatogenesis associated glutamate (E)-rich protein 4C, opposite strand transcript	pseudo
4930572O03Rik	1	1.03	1.11	2.64	2.65	2.74	1.00	1.04	ns	ns	up	381715	unknown	-		5	spermatogenesis associated glutamate (E)-rich protein pseudogene	pseudo
Mcf2l	1	0.52	0.59	0.59	0.56	0.54	0.96	0.92	dn	ns	ns	17207	other	C130040G20Rik|Dbs|Ost|mKIAA0362		8	mcf.2 transforming sequence-like	protein-coding
Adamts9	1	0.54	0.59	0.55	0.51	0.53	0.93	0.97	dn	ns	dn	101401	matrix	1810011L16Rik|8430403M15Rik|AW743315|E030027K14Rik		6	a disintegrin-like and metallopeptidase (reprolysin type) with thrombospondin type 1 motif, 9	protein-coding
Rasgef1b	1	0.56	0.65	0.49	0.53	0.55	1.07	1.13	dn	ns	dn	320292	other	4732452O09Rik|GPIgamma4|Gpig4		5	RasGEF domain family, member 1B	protein-coding
Atf3	1	0.59	0.52	0.58	0.63	0.56	1.09	0.96	dn	ns	ns	11910	other	LRG-21		1	activating transcription factor 3	protein-coding
Apold1	1	0.54	0.55	0.49	0.59	0.54	1.20	1.09	dn	ns	dn	381823	unknown	Gm1075		6	apolipoprotein L domain containing 1	protein-coding
Lrrc16a	1	0.63	0.64	0.53	0.60	0.64	1.15	1.22	dn	ns	dn	68732	unknown	1110037D04Rik|AI425970|CARMIL|CARML1|D130057M20|Lrrc16		13	leucine rich repeat containing 16A	protein-coding
Akap13	1	0.60	0.62	0.54	0.57	0.66	1.04	1.21	dn	ns	dn	75547	other	1700026G02Rik|5730522G15Rik|5830460E08Rik|AKAP-Lbc|BRX|Ht31|LBC|PROTO-LB|PROTO		7	A kinase (PRKA) anchor protein 13	protein-coding
Nedd4l	1	0.62	0.58	0.54	0.55	0.60	1.01	1.10	dn	ns	dn	83814	other	1300012C07Rik|Nedd4-2|Nedd4b		18	neural precursor cell expressed, developmentally down-regulated gene 4-like	protein-coding
Nfil3	1	0.66	0.69	0.53	0.54	0.71	1.02	1.34	dn	ns	dn	18030	other	AV225605|E4BP4		13	nuclear factor, interleukin 3, regulated	protein-coding
Trib2	1	0.59	0.68	0.64	0.58	0.59	0.91	0.92	dn	ns	dn	217410	other	AW319517|TRB-2|TRB2		12	tribbles pseudokinase 2	protein-coding
Gbe1	1	0.57	0.64	0.58	0.63	0.55	1.09	0.94	dn	ns	dn	74185	other	2310045H19Rik|2810426P10Rik|D16Ertd536e		16	glucan (1,4-alpha-), branching enzyme 1	protein-coding
Ccdc85a	1	0.57	0.68	0.61	0.54	0.51	0.87	0.82	dn	ns	dn	216613	unknown	-		11	coiled-coil domain containing 85A	protein-coding
Slc10a6	1	0.54	0.70	0.67	0.54	0.67	0.80	1.00	dn	ns	ns	75750	other	8430417G17Rik|C78479|Soat		5	solute carrier family 10 (sodium/bile acid cotransporter family), member 6	protein-coding
Klf15	1	0.54	0.62	0.68	0.55	0.63	0.81	0.92	dn	ns	ns	66277	other	1810013I09Rik|AV048136|AW494632|CKLF|KKLF		6	Kruppel-like factor 15	protein-coding
Tmem252	1	0.52	0.65	0.64	0.63	0.66	0.99	1.04	dn	ns	dn	226040	unknown	E030010A14Rik		19	transmembrane protein 252	protein-coding
E030003E18Rik	1	0.48	0.64	0.52	0.47	0.45	0.91	0.88	dn	ns	dn	320092	unknown	1700127G21Rik		19	RIKEN cDNA E030003E18 gene	ncRNA
Adamts15	1	0.56	0.53	0.52	0.45	0.46	0.87	0.89	dn	ns	dn	235130	matrix	-		9	a disintegrin-like and metallopeptidase (reprolysin type) with thrombospondin type 1 motif, 15	protein-coding
Mettl21e	1	0.40	0.54	0.54	0.40	0.48	0.73	0.88	dn	ns	dn	403183	unknown	4832428D23Rik|Gm991		1	methyltransferase like 21E	protein-coding
S1pr1	1	0.53	0.52	0.59	0.50	0.58	0.85	0.98	dn	ns	dn	13609	other	AI849002|Edg1|Lpb1|S1p|S1p1		3	sphingosine-1-phosphate receptor 1	protein-coding
Foxo3	1	0.47	0.55	0.54	0.46	0.56	0.85	1.03	dn	ns	dn	56484	other	1110048B16Rik|2010203A17Rik|C76856|FKHRL1|Fkhr2|Foxo3a		10	forkhead box O3	protein-coding
Cdkn1a	1	0.45	0.47	0.59	0.46	0.56	0.78	0.95	dn	ns	dn	12575	other	CAP20|CDKI|CIP1|Cdkn1|P21|SDI1|Waf1|mda6|p21Cip1|p21WAF		17	cyclin-dependent kinase inhibitor 1A (P21)	protein-coding
Snrk	1	0.68	0.72	0.66	0.66	0.69	1.01	1.05	dn	ns	dn	20623	other	2010012F07Rik|AI448042|AW547029|E030034B15|R74830|mKIAA0096		9	SNF related kinase	protein-coding
Ccdc141	1	0.68	0.78	0.64	0.62	0.66	0.96	1.03	ns	ns	dn	545428	unknown	2610301F02Rik|CAMDI|ENSMUSG00000075261		2	coiled-coil domain containing 141	protein-coding
Lnx2	1	0.66	0.76	0.66	0.63	0.75	0.95	1.13	dn	ns	dn	140887	other	9630046H24|AW209022		5	ligand of numb-protein X 2	protein-coding
5830436I19Rik	1	0.59	0.75	0.65	0.70	0.62	1.08	0.95	dn	ns	ns	319946	unknown	_	_		_	discontinued
Rapgef4	1	0.73	0.74	0.66	0.64	0.56	0.97	0.85	ns	ns	dn	56508	other	1300003D15Rik|5730402K07Rik|6330581N18Rik|EPAC 2|Epac-2|Epac2|mKIAA4040		2	Rap guanine nucleotide exchange factor (GEF) 4	protein-coding
Adamtsl2	1	0.68	0.82	0.64	0.54	0.60	0.85	0.94	dn	ns	dn	77794	matrix	A930008K15Rik|tcp-1		2	ADAMTS-like 2	protein-coding
Epb41	1	0.62	0.73	0.64	0.62	0.67	0.97	1.05	dn	ns	dn	269587	unknown	4.1R|AI415518|D4Ertd442e|Elp-1|Elp1|Epb4.1|mKIAA4056		4	erythrocyte membrane protein band 4.1	protein-coding
Cdc42ep4	1	0.66	0.67	0.67	0.63	0.64	0.94	0.96	dn	ns	ns	56699	other	1500041M20Rik|Borg4|CEP4		11	CDC42 effector protein (Rho GTPase binding) 4	protein-coding
Mpp3	1	0.60	0.65	0.66	0.60	0.69	0.92	1.06	dn	ns	ns	13384	unknown	6430514B01|Dlgh3		11	membrane protein, palmitoylated 3 (MAGUK p55 subfamily member 3)	protein-coding
Rhob	1	0.65	0.64	0.58	0.65	0.67	1.11	1.15	dn	ns	ns	11852	other	AA017882|Arh6|Arhb		12	ras homolog family member B	protein-coding
9430076C15Rik	1	0.66	0.67	0.62	0.65	0.68	1.05	1.09	dn	ns	dn	320189	other	-		6	RIKEN cDNA 9430076C15 gene	ncRNA
Klf7	1	0.66	0.68	0.63	0.59	0.64	0.94	1.01	dn	ns	dn	93691	other	9830124P08Rik		1	Kruppel-like factor 7 (ubiquitous)	protein-coding
9330159F19Rik	1	0.65	0.67	0.63	0.57	0.61	0.90	0.98	dn	ns	dn	212448	unknown	-		10	RIKEN cDNA 9330159F19 gene	protein-coding
Lmcd1	1	0.70	0.68	0.66	0.57	0.62	0.86	0.94	ns	ns	dn	30937	other	AW455500|AW553074|dyxin		6	LIM and cysteine-rich domains 1	protein-coding
Adgrl3	1	0.63	0.60	0.67	0.67	0.76	1.00	1.12	dn	ns	ns	319387	unknown	5430402I23Rik|CIRL-3|D130075K09Rik|Gm1379|LEC3|Lphn3|mKIAA0768		5	adhesion G protein-coupled receptor L3	protein-coding
Kcnj15	1	0.65	0.68	0.70	0.62	0.78	0.89	1.11	dn	ns	ns	16516	other	4930414N08Rik|AI182284|AI267127|IRKK|Kir4.2		16	potassium inwardly-rectifying channel, subfamily J, member 15	protein-coding
Spsb1	1	0.65	0.66	0.72	0.68	0.71	0.94	0.99	dn	ns	ns	74646	other	1110014L01Rik|4930422J18Rik|AI596360|AI854583|SSB-1|SSB1		4	splA/ryanodine receptor domain and SOCS box containing 1	protein-coding
Rgs9	1	0.63	0.65	0.72	0.66	0.72	0.92	1.01	dn	ns	ns	19739	other	RGS9-1|Rgs9-2		11	regulator of G-protein signaling 9	protein-coding
Eva1c	1	0.63	0.65	0.77	0.68	0.73	0.88	0.94	dn	ns	ns	70967	unknown	1700092M14Rik|4931408A02Rik|Fam176c		16	eva-1 homolog C (C. elegans)	protein-coding
Dusp16	1	0.66	0.66	0.70	0.62	0.69	0.88	0.99	dn	ns	ns	70686	other	3830417M17Rik|AW558566|D6Ertd213e|MKP-7|MKP7|Mkpm		6	dual specificity phosphatase 16	protein-coding
Acta2	1	0.64	0.67	0.75	0.64	0.68	0.85	0.91	dn	ns	ns	11475	other	0610041G09Rik|Actvs|SMalphaA|a-SMA|alphaSMA		19	actin, alpha 2, smooth muscle, aorta	protein-coding
Klhl2	1	0.69	0.70	0.75	0.62	0.69	0.82	0.92	ns	dn	dn	77113	other	6030411N21Rik|ABP-KELCH|AU020744|Mav		8	kelch-like 2, Mayven	protein-coding
Creb5	1	0.63	0.61	0.71	0.65	0.68	0.91	0.96	dn	ns	ns	231991	other	Crebpa|D430026C09Rik		6	cAMP responsive element binding protein 5	protein-coding
Bcl2l1	1	0.61	0.62	0.69	0.63	0.63	0.91	0.91	dn	ns	ns	12048	other	Bcl(X)L|Bcl-XL|Bcl2l|BclX|bcl-x|bcl2-L-1		2	BCL2-like 1	protein-coding
AA986860	1	0.57	0.68	0.74	0.62	0.65	0.83	0.87	dn	ns	ns	212439	unknown	E030025M07|Sarg		1	expressed sequence AA986860	protein-coding
Il1r2	1	0.52	0.55	0.75	0.56	0.64	0.75	0.85	dn	ns	ns	16178	other	CD121b|Il1r-2		1	interleukin 1 receptor, type II	protein-coding
Gm3579	1	0.51	0.57	0.82	0.59	0.66	0.72	0.80	dn	ns	ns	100041932	gene model	H3097D08		4	predicted gene 3579	other
Rapgef5	1	0.61	0.59	0.75	0.51	0.64	0.69	0.86	dn	ns	ns	217944	other	4932413M22|C86120|D030051B22Rik|GFR|Mrgef|mKIAA0277|mmr-gef		12	Rap guanine nucleotide exchange factor (GEF) 5	protein-coding
Pmvk	1	0.58	0.62	0.78	0.54	0.68	0.70	0.87	dn	ns	ns	68603	other	1110011E12Rik|2900002L22Rik|AV001327|PMK|PMKA|PMKASE		3	phosphomevalonate kinase	protein-coding
Krtap17-1	1	0.68	0.59	0.74	0.62	0.54	0.83	0.72	dn	ns	ns	77914	other	A030006P16Rik		11	keratin associated protein 17-1	protein-coding
A130040M12Rik	1	0.62	0.61	0.82	0.54	0.55	0.66	0.68	dn	ns	ns	319269	unknown	CA782090|H3053C11|VL30		11	RIKEN cDNA A130040M12 gene	other
Fkbp14	1	0.60	0.62	0.90	0.62	0.62	0.69	0.69	dn	ns	ns	231997	other	BC029109|FKBP-14|FKBP22		6	FK506 binding protein 14	protein-coding
Fam46b	1	0.60	0.60	0.88	0.64	0.63	0.73	0.72	dn	ns	ns	100342	unknown	4732473B16Rik|AU045678|AU046069		4	family with sequence similarity 46, member B	protein-coding
Gm6016	1	0.60	0.58	0.92	0.65	0.65	0.71	0.71	dn	ns	ns	547034	gene model	_	_		_	discontinued
Gm4080	1	0.56	0.59	0.88	0.61	0.63	0.69	0.72	dn	ns	ns	100042868	gene model	_	_		_	discontinued
Ctla2b	1	0.57	0.63	0.94	0.65	0.69	0.69	0.74	dn	ns	ns	13025	other	AW545568|Ctla-2b		13	cytotoxic T lymphocyte-associated protein 2 beta	protein-coding
Bcar3	1	0.59	0.61	0.87	0.57	0.65	0.66	0.75	dn	dn	ns	29815	other	AI131758|AND-34		3	breast cancer anti-estrogen resistance 3	protein-coding
Pxdc1	1	0.61	0.62	0.88	0.59	0.69	0.67	0.78	dn	ns	ns	66895	unknown	1300014I06Rik		13	PX domain containing 1	protein-coding
Sgms1	1	0.63	0.65	0.87	0.59	0.61	0.68	0.71	dn	dn	ns	208449	other	9530058O11Rik|AI841905|C80702|Mob|Sms1|Sor1|Tmem23		19	sphingomyelin synthase 1	protein-coding
Gm3153	1	0.51	0.51	0.85	0.61	0.61	0.71	0.72	dn	ns	ns	100041127	gene model	_	_		_	discontinued
Gm2948	1	0.53	0.50	0.89	0.64	0.62	0.72	0.70	dn	ns	ns	100040753	gene model	_	_		_	discontinued
Gm3061	1	0.49	0.50	0.92	0.62	0.62	0.67	0.68	dn	ns	ns	100040952	gene model	_	_		_	discontinued
F3	1	0.56	0.54	0.87	0.65	0.56	0.74	0.65	dn	ns	ns	14066	other	AA409063|CD142|Cf-3|Cf3|TF		3	coagulation factor III	protein-coding
Gm2627	1	0.53	0.49	0.80	0.46	0.48	0.57	0.61	dn	dn	ns	100040148	gene model	_	_		_	discontinued
Gm2194	1	0.55	0.49	0.85	0.47	0.53	0.55	0.63	dn	ns	ns	100039378	gene model	_	_		_	discontinued
Maff	1	0.52	0.50	0.78	0.51	0.57	0.66	0.72	dn	dn	ns	17133	other	-		15	v-maf musculoaponeurotic fibrosarcoma oncogene family, protein F (avian)	protein-coding
Klhl29	1	0.84	0.87	1.34	0.81	0.92	0.61	0.69	ns	dn	ns	208439	unknown	A230106N14Rik|Gm68|Kbtbd9|mKIAA1921		12	kelch-like 29	protein-coding
Fmnl2	1	0.85	0.93	1.31	0.81	0.95	0.62	0.73	ns	dn	ns	71409	other	5430425K04Rik|Man		2	formin-like 2	protein-coding
Gadd45b	1	0.94	0.82	1.27	0.78	0.79	0.62	0.63	ns	dn	ns	17873	other	AI323528|Myd118		10	growth arrest and DNA-damage-inducible 45 beta	protein-coding
Rpa1	1	0.71	0.88	1.08	0.71	0.95	0.65	0.88	ns	dn	ns	68275	other	5031405K23Rik|70kDa|AA589576|AW557552|RF-A|RP-A|Rpa		11	replication protein A1	protein-coding
Krt15	1	0.75	0.73	1.22	0.84	0.70	0.69	0.57	ns	dn	ns	16665	matrix	AI528832|K15|Krt1-15		11	keratin 15	protein-coding
Klf11	1	0.71	0.80	0.96	0.72	0.61	0.75	0.63	ns	dn	ns	194655	other	9830142A17|D12Ertd427e|Tieg2|Tieg2b|Tieg3		12	Kruppel-like factor 11	protein-coding
Hspa1a	1	0.91	0.61	0.54	0.80	1.27	1.49	2.35	ns	ns	dn	193740	other	Hsp70-3|Hsp70.3|Hsp72|hsp68|hsp70A1		17	heat shock protein 1A	protein-coding
Gm7816	1	0.79	0.81	0.79	0.81	1.19	1.03	1.51	ns	up	ns	665839	gene model	EG665839		5	DnaJ (Hsp40) homolog, subfamily A, member 1 pseudogene	pseudo
Herpud1	1	0.62	0.62	0.83	0.65	0.80	0.78	0.97	dn	ns	ns	64209	other	Herp|Mifl|SUP		8	homocysteine-inducible, endoplasmic reticulum stress-inducible, ubiquitin-like domain member 1	protein-coding
Crem	1	0.61	0.65	0.79	0.62	0.75	0.78	0.95	dn	ns	ns	12916	other	ICER|ICERI		18	cAMP responsive element modulator	protein-coding
Plxna2	1	0.68	0.70	0.93	0.60	0.78	0.65	0.83	ns	dn	ns	18845	other	2810428A13Rik|AA589422|AW457381|OCT|PlexA2|Plxn2|mKIAA0463		1	plexin A2	protein-coding
Trp53inp1	1	0.61	0.66	0.89	0.74	0.78	0.83	0.88	dn	ns	ns	60599	other	2700057G22Rik|SIP|SIP18|SIP27|Stinp|Teap		4	transformation related protein 53 inducible nuclear protein 1	protein-coding
Pex11a	1	0.68	0.67	0.87	0.73	0.80	0.84	0.92	dn	ns	ns	18631	other	PEX11alpha		7	peroxisomal biogenesis factor 11 alpha	protein-coding
Ctla2a	1	0.66	0.70	1.03	0.76	0.79	0.74	0.77	dn	ns	ns	13024	other	Ctla-2a		13	cytotoxic T lymphocyte-associated protein 2 alpha	protein-coding
Pfkfb3	1	0.63	0.66	0.77	0.73	0.66	0.94	0.85	dn	ns	ns	170768	other	E330010H22Rik|iPFK-2|uPFK-2		2	6-phosphofructo-2-kinase/fructose-2,6-biphosphatase 3	protein-coding
Adrb2	1	0.63	0.76	0.78	0.70	0.68	0.90	0.87	dn	ns	ns	11555	other	Adrb-2|Badm|Gpcr7		18	adrenergic receptor, beta 2	protein-coding
Mbd1	1	0.62	0.69	0.81	0.66	0.70	0.81	0.87	dn	ns	ns	17190	other	Cxxc3|PCM1		18	methyl-CpG binding domain protein 1	protein-coding
Medag	1	0.66	0.68	0.85	0.68	0.71	0.80	0.84	dn	ns	ns	70717	unknown	6330406I15Rik|Awms3|MEDA-4|Meda4|mAWMS3		5	mesenteric estrogen dependent adipogenesis	protein-coding
Gm2563	1	0.66	0.68	0.84	0.67	0.66	0.81	0.79	dn	ns	ns	100040034	gene model	_	_		_	discontinued
Gm4499	1	0.63	0.60	0.89	0.71	0.67	0.80	0.75	dn	ns	ns	100043524	gene model	_	_		_	discontinued
Gm2015	1	0.61	0.63	0.87	0.71	0.68	0.82	0.78	dn	ns	ns	100039039	gene model	_	_		_	discontinued
Tnfrsf12a	1	0.69	0.60	0.91	0.73	0.61	0.79	0.67	dn	dn	ns	27279	other	AI255180|C87282|Fn14|HPIP|TWEAK-R|TweakR		17	tumor necrosis factor receptor superfamily, member 12a	protein-coding
Gm3430	1	0.66	0.66	0.93	0.71	0.73	0.76	0.78	dn	ns	ns	100041612	gene model	_	_		_	discontinued
Gm2031	1	0.66	0.69	0.91	0.70	0.70	0.78	0.77	dn	ns	ns	100039066	gene model	_	_		_	discontinued
Gm3459	1	0.66	0.65	0.92	0.75	0.73	0.82	0.80	dn	ns	ns	100041663	gene model	_	_		_	discontinued
Gm3009	1	0.63	0.65	0.93	0.70	0.68	0.75	0.74	dn	ns	ns	100040864	gene model	_	_		_	discontinued
Gm13105	1	0.65	0.65	0.93	0.70	0.70	0.75	0.75	dn	ns	ns	100041144	gene model	OTTMUSG00000010436		4	predicted gene 13105	protein-coding
Gm3707	1	0.65	0.64	0.90	0.69	0.68	0.77	0.76	dn	ns	ns	100042177	gene model	_	_		_	discontinued
Gm15035	1	0.66	0.65	0.91	0.69	0.69	0.76	0.76	dn	ns	ns	100040295	gene model	OTTMUSG00000018820	X		predicted gene 15035	protein-coding
Gm3885	1	0.65	0.63	0.91	0.69	0.69	0.76	0.76	dn	ns	ns	100042523	gene model	_	_		_	discontinued
Tsc22d3	1	0.31	0.43	0.46	0.35	0.42	0.76	0.91	dn	ns	dn	14605	other	DIP|Dsip1|Gilz|TSC-22R|Tilz3	X		TSC22 domain family, member 3	protein-coding
Nr4a3	1	0.32	0.34	0.55	0.42	0.36	0.76	0.64	dn	ns	ns	18124	immune (other)	AI573420|CHN|CSMF|MINOR|NOR-1|Nor1|TEC		4	nuclear receptor subfamily 4, group A, member 3	protein-coding
Per1	1	0.31	0.58	0.40	0.47	0.43	1.17	1.08	dn	ns	ns	18626	other	Per|m-rigui|mPer1		11	period circadian clock 1	protein-coding
Angptl4	1	0.21	0.43	0.62	0.29	0.30	0.46	0.48	dn	ns	ns	57875	matrix	Arp4|Bk89|Fiaf|Hfarp|Ng27|Pgar|Pgarg|Pp1158		17	angiopoietin-like 4	protein-coding
Errfi1	1	0.25	0.32	0.28	0.28	0.26	1.00	0.95	dn	ns	ns	74155	other	1300002F13Rik|AI788755|Mig-6|Mig6|RALT		4	ERBB receptor feedback inhibitor 1	protein-coding
Zbtb16	1	0.20	0.24	1.18	0.23	0.41	0.19	0.35	dn	dn	ns	235320	other	AI467657|PLZF|Zfp145|lu		9	zinc finger and BTB domain containing 16	protein-coding
Gm4569	1	0.45	0.44	0.77	0.40	0.46	0.52	0.59	dn	dn	ns	100043650	gene model	_	_		_	discontinued
Gm2251	1	0.48	0.44	0.78	0.42	0.46	0.54	0.59	dn	dn	ns	100039464	gene model	_	_		_	discontinued
Gm3245	1	0.44	0.44	0.78	0.42	0.47	0.53	0.60	dn	dn	ns	100041274	gene model	_	_		_	discontinued
Fkbp5	1	0.42	0.44	0.79	0.42	0.47	0.54	0.60	dn	dn	ns	14229	other	D17Ertd592e|Dit1|FKBP-5|FKBP51		17	FK506 binding protein 5	protein-coding
Thbs1	1	0.51	0.42	0.65	0.46	0.40	0.71	0.62	dn	dn	ns	21825	other	TSP-1|TSP1|Thbs-1|tbsp1		2	thrombospondin 1	protein-coding
Gm3651	1	0.43	0.40	0.80	0.39	0.42	0.49	0.52	dn	dn	ns	100042075	gene model	_	_		_	discontinued
Gm2326	1	0.44	0.41	0.76	0.40	0.44	0.52	0.58	dn	dn	ns	100039583	gene model	_	_		_	discontinued
Gm4148	1	0.41	0.40	0.75	0.36	0.41	0.48	0.54	dn	dn	ns	100042984	gene model	_	_		_	discontinued
Gm3593	1	0.41	0.39	0.76	0.37	0.40	0.49	0.53	dn	dn	ns	100041962	gene model	_	_		_	discontinued
Map3k6	1	0.39	0.42	0.77	0.38	0.47	0.50	0.61	dn	dn	ns	53608	other	Ask2|MAPKKK6|MEKK6		4	mitogen-activated protein kinase kinase kinase 6	protein-coding
Rgcc	1	0.37	0.42	0.68	0.37	0.44	0.55	0.64	dn	ns	ns	66214	immune (other)	1190002H23Rik|Rgc-32|Rgc32		14	regulator of cell cycle	protein-coding
Pdk4	1	0.38	0.50	0.81	0.49	0.57	0.60	0.70	dn	ns	ns	27273	other	AV005916		6	pyruvate dehydrogenase kinase, isoenzyme 4	protein-coding
Fam107a	1	0.33	0.45	0.93	0.37	0.48	0.40	0.52	dn	dn	ns	268709	unknown	DRR1		14	family with sequence similarity 107, member A	protein-coding
Nrcam	1	0.92	0.96	0.60	0.60	0.60	1.00	1.01	ns	ns	dn	319504	other	Bravo|C030017F07Rik|C130076O07Rik|mKIAA0343		12	neuronal cell adhesion molecule	protein-coding
Edil3	1	0.93	1.00	0.65	0.58	0.58	0.89	0.89	ns	ns	dn	13612	other	Del-1|Del1		13	EGF-like repeats and discoidin I-like domains 3	protein-coding
Peg3	1	0.96	0.93	0.64	0.62	0.63	0.96	0.98	ns	ns	dn	18616	other	AL022617|ASF-1|End4|Gcap4|Pw1|Zfp102|mKIAA0287		7	paternally expressed 3	protein-coding
Fbn1	1	0.97	0.95	0.62	0.64	0.62	1.03	0.99	ns	ns	dn	14118	matrix	AI536462|B430209H23|Fib-1|Tsk		2	fibrillin 1	protein-coding
Postn	1	1.00	0.92	0.63	0.61	0.59	0.96	0.93	ns	ns	dn	50706	matrix	A630052E07Rik|AI747096|OSF-2|Osf2|PLF|PN		3	periostin, osteoblast specific factor	protein-coding
Gucy1a3	1	1.00	0.92	0.62	0.60	0.62	0.98	1.00	ns	ns	dn	60596	other	1200016O07Rik|sGC-alpha1		3	guanylate cyclase 1, soluble, alpha 3	protein-coding
Pde5a	1	1.02	0.88	0.61	0.70	0.56	1.13	0.91	ns	ns	dn	242202	other	Cgbpde|Cn5n|PDE5a2|Pde5|Pde5a1		3	phosphodiesterase 5A, cGMP-specific	protein-coding
Col1a1	1	1.05	0.97	0.63	0.66	0.57	1.05	0.90	ns	ns	dn	12842	matrix	Col1a-1|Cola-1|Cola1|Mov-13|Mov13		11	collagen, type I, alpha 1	protein-coding
Sparc	1	1.03	0.90	0.64	0.62	0.53	0.97	0.83	ns	ns	dn	20692	matrix	BM-40|ON		11	secreted acidic cysteine rich glycoprotein	protein-coding
Bex1	1	1.00	0.96	0.65	0.62	0.55	0.94	0.84	ns	ns	dn	19716	other	AA407839|AU018692|Bex4|Rex3	X		brain expressed X-linked 1	protein-coding
Smarca1	1	0.94	0.97	0.65	0.66	0.64	1.01	1.00	ns	ns	dn	93761	other	5730494M04Rik|Snf2l	X		SWI/SNF related, matrix associated, actin dependent regulator of chromatin, subfamily a, member 1	protein-coding
Lrch2	1	0.93	1.00	0.66	0.64	0.66	0.97	1.00	ns	ns	dn	210297	unknown	AI316885|mKIAA1495	X		leucine-rich repeats and calponin homology (CH) domain containing 2	protein-coding
Robo2	1	0.92	0.97	0.66	0.69	0.67	1.04	1.02	ns	ns	dn	268902	other	2600013A04Rik|9430089E08Rik|BB097918|D230004I22Rik|mKIAA1568		16	roundabout guidance receptor 2	protein-coding
Frem1	1	0.97	0.97	0.66	0.73	0.65	1.11	0.98	ns	ns	dn	329872	matrix	BC037594|D430009N09|D630008K06|crf11|eye<m02Jus>|eyes2|heb|qbrick		4	Fras1 related extracellular matrix protein 1	protein-coding
Lef1	1	1.02	1.07	0.61	0.69	0.67	1.13	1.09	ns	ns	dn	16842	immune (other)	3000002B05|AI451430|Lef-1		3	lymphoid enhancer binding factor 1	protein-coding
Itm2a	1	0.94	1.08	0.61	0.61	0.70	1.01	1.16	ns	ns	dn	16431	other	AW108051|Bricd2a|E25|Itm2	X		integral membrane protein 2A	protein-coding
Daam2	1	0.86	0.98	0.58	0.61	0.73	1.05	1.26	ns	ns	dn	76441	other	2310016D11Rik|AI843643|AW557870		17	dishevelled associated activator of morphogenesis 2	protein-coding
Mgp	1	1.10	0.98	0.62	0.85	0.68	1.38	1.09	ns	ns	dn	17313	other	Mglap		6	matrix Gla protein	protein-coding
Col1a2	1	1.15	1.03	0.57	0.72	0.56	1.27	0.98	ns	ns	dn	12843	matrix	AA960264|AI325291|Col1a-2|Cola-2|Cola2|oim		6	collagen, type I, alpha 2	protein-coding
Tmem200a	1	1.14	1.14	0.63	0.77	0.76	1.21	1.20	ns	ns	dn	77220	other	C030003D03Rik		10	transmembrane protein 200A	protein-coding
Cttnbp2	1	1.11	1.22	0.59	0.72	0.76	1.22	1.29	ns	ns	dn	30785	other	3010022N24Rik|4732477G22Rik|6430526E05|9130022E09Rik|AU040881|Cortbp2|ORF4|mKIAA1		6	cortactin binding protein 2	protein-coding
Tnc	1	0.78	0.85	0.61	0.64	0.62	1.04	1.02	ns	ns	dn	21923	matrix	AI528729|C130033P17Rik|Hxb|TN|TN-C|Ten|cytotactin|tenascin-C		4	tenascin C	protein-coding
Grb10	1	0.76	0.87	0.64	0.61	0.69	0.94	1.07	ns	ns	dn	14783	other	5730571D09Rik|AI325020|Meg1|mKIAA0207		11	growth factor receptor bound protein 10	protein-coding
Spon2	1	0.85	0.83	0.60	0.70	0.63	1.16	1.04	ns	ns	dn	100689	matrix	2310045I24Rik|AI504350|M-spondin|Mindin|Mspondin		5	spondin 2, extracellular matrix protein	protein-coding
Serpinh1	1	0.86	0.84	0.65	0.67	0.60	1.03	0.91	ns	ns	dn	12406	other	BERF-1|Cbp1|Cbp2|Hsp47|J6|Serpinh2|gp46		7	serine (or cysteine) peptidase inhibitor, clade H, member 1	protein-coding
Fam19a1	1	0.90	0.83	0.60	0.59	0.57	0.99	0.95	ns	ns	dn	320265	other	C630007B19Rik|FAM19A1 TAFA-1|TAFA1|Tafa-1		6	family with sequence similarity 19, member A1	protein-coding
Fam162b	1	0.88	0.84	0.61	0.58	0.56	0.95	0.92	ns	ns	dn	77296	other	9430073N08Rik		10	family with sequence similarity 162, member B	protein-coding
Col4a1	1	0.86	0.78	0.61	0.60	0.53	0.98	0.87	ns	ns	dn	12826	matrix	Bru|Col4a-1|Raw|Svc		8	collagen, type IV, alpha 1	protein-coding
Meg3	1	0.84	0.89	0.64	0.58	0.56	0.91	0.88	ns	ns	dn	17263	unknown	2900016C05Rik|3110050O07Rik|6330408G06Rik|AI425946|AW108224|D12Bwg1266e|Gtl2|R7475		12	maternally expressed 3	ncRNA
Nrep	1	1.01	1.06	0.50	0.56	0.49	1.13	0.99	ns	ns	dn	27528	unknown	AI325076|D0H4S114|Harp|P311|PTZ17|SEZ17		18	neuronal regeneration related protein	protein-coding
Col5a2	1	1.05	1.02	0.51	0.60	0.53	1.18	1.05	ns	ns	dn	12832	matrix	1110014L14Rik		1	collagen, type V, alpha 2	protein-coding
Col3a1	1	1.06	1.02	0.43	0.55	0.47	1.27	1.08	ns	ns	dn	12825	matrix	AW550625|Col3a-1|Tsk-2|Tsk2		1	collagen, type III, alpha 1	protein-coding
Nrxn1	1	0.96	1.01	0.56	0.56	0.55	1.00	0.99	ns	ns	dn	18189	other	1700062G21Rik|9330127H16Rik|A230068P09Rik|mKIAA0578		17	neurexin I	protein-coding
C430049B03Rik	1	0.92	1.01	0.52	0.49	0.57	0.95	1.10	ns	ns	dn	72575	unknown	2700063P19Rik|9430052C07Rik|mir-322|mir-351|mir-503	X		RIKEN cDNA C430049B03 gene	ncRNA
Mboat2	1	0.97	0.97	0.50	0.50	0.50	1.01	1.00	ns	ns	dn	67216	other	2810049G06Rik|AU022889|AW547221|LPCAT4|Moact2|Oact2		12	membrane bound O-acyltransferase domain containing 2	protein-coding
Cyp1a1	1	1.23	1.49	0.34	0.56	0.44	1.64	1.29	ns	ns	dn	13076	other	AHH|AHRR|CP11|Cyp1a2|P450-1		9	cytochrome P450, family 1, subfamily a, polypeptide 1	protein-coding

Legend table S1
YM0, young, mock-inoculated
YM2, young, two days post RSV-inoculation
YM5, young, five days post RSV-inoculation
OM0, old, mock-inoculated
OM2, old, two days post RSV-inoculation
OM5, old, five days post RSV-inoculation

ns, not significant
up, significantly upregulated expression
dn, significantly downregulated expression

Table S2. Predicted upstream regulators (p<1E-10)

RSV young	Status	Fold change	RSV old	Status	Fold change
poly rI:rC-RNA	Activated	6.04	IFNG	Activated	4.45
IFNA2	Activated	5.34	LPS	Activated	4.09
IRF7	Activated	5.34	IFNA2	Activated	3.90
IRF3	Activated	5.31	IRF7	Activated	3.78
IFNG	Activated	5.29	STAT1	Activated	3.75
LPS	Activated	5.28	IRF3	Activated	3.64
STAT1	Activated	5.15	poly rI:rC-RNA	Activated	3.59
Ifnar	Activated	4.99	PRL	Activated	3.56
PRL	Activated	4.56	TLR3	Activated	3.53
TLR3	Activated	4.37	TLR9	Activated	3.50
IFN Beta	Activated	4.28	Interferon alpha	Activated	3.42
IFNL1	Activated	4.22	IFN Beta	Activated	3.39
Interferon alpha	Activated	4.20	IFNL1	Activated	3.25
IFNB1	Activated	4.07	Ifnar	Activated	3.25
IRF5	Activated	3.90	TLR4	Activated	3.22
TNF	Activated	3.89	IFNB1	Activated	3.21
TGM2	Activated	3.86	SAMSN1	Activated	3.16
TLR9	Activated	3.85	TICAM1	Activated	3.11
IRF1	Activated	3.76	IRF5	Activated	3.09
SASH1	Activated	3.74	DOCK8	Activated	3.00
SAMSN1	Activated	3.74	SASH1	Activated	3.00
E. coli B4 LPS	Activated	3.74	stallimycin	Activated	2.96
TICAM1	Activated	3.69	bromodeoxyuridine	Activated	2.96
MAVS	Activated	3.67	*TLR7*	Activated	2.95
IFNAR1	Activated	3.66	IRF1	Activated	2.92
Ifn	Activated	3.65	Map3k7	Activated	2.81
IL21	Activated	3.63	IFN alpha/beta	Activated	2.78
APP	Activated	3.61	Ifn	Activated	2.78
DDX58	Activated	3.51	MAVS	Activated	2.76
TLR4	Activated	3.50	DDX58	Activated	2.74
IFN type 1	Activated	3.37	IFNA1/IFNA13	Activated	2.72
IFNA1/IFNA13	Activated	3.37	oblimersen	Activated	2.65
DOCK8	Activated	3.32	TMEM173	Activated	2.63
stallimycin	Activated	3.29	FZD9	Activated	2.62
bromodeoxyuridine	Activated	3.29	*IKBKE*	Activated	2.60
IFN alpha/beta	Activated	3.25	CGAS	Activated	2.59
tretinoin	Activated	3.25	IFNAR1	Activated	2.59
TMEM173	Activated	3.13	STAT2	Activated	2.59
MYD88	Activated	3.02	IL21	Activated	2.53
oblimersen	Activated	3.00	PARP9	Activated	2.43
FADD	Activated	2.99	IFNA4	Activated	2.36
FZD9	Activated	2.97	NFATC2	Activated	2.34
Map3k7	Activated	2.95	TBK1	Activated	2.24
CGAS	Activated	2.95	TRIM24	Inhibited	-3.46
JAK	Activated	2.83	ACKR2	Inhibited	-3.32
STAT2	Activated	2.77	PTGER4	Inhibited	-3.27
IFIH1	Activated	2.77	SIRT1	Inhibited	-3.26
NFATC2	Activated	2.69	MAPK1	Inhibited	-2.67
PARP9	Activated	2.63	Irgm1	Inhibited	-2.65

IFNA4	Activated	2.55	*STAT3*	Inhibited	-2.63
TBK1	Activated	2.45	SOCS1	Inhibited	-2.62
TRIM24	Inhibited	-4.70			
SIRT1	Inhibited	-4.38			
ACKR2	Inhibited	-4.36			
MAPK1	Inhibited	-4.15			
SOCS1	Inhibited	-3.92			
PTGER4	Inhibited	-3.90			
Irgm1	Inhibited	-3.46			
NKX2-3	Inhibited	-3.36			
IL1RN	Inhibited	-2.99			
USP18	Inhibited	-2.62			
DNASE2	Inhibited	-2.60			
IRF4	Inhibited	-2.23			

Positive values depict z-score as indicator of activation of upstream regulator, negative values depict z-score as indicator of inhibition of upstream regulator. Predicted regulators written in italics are only found regulated by z-score >2 and $P<1x10^{-10}$ *at old age.*

TABLE S3. Validation

Microarray

Symbol	Pathway	YM0/YM0	YR2/YM0	YR5/YM0	OM0/YM0	OR2/YM0	OR5/YM0	OR2/OM0	OR5/OM0	Gene name
B2m	antigen proc&pres	1.00	1.68	1.55	1.32	2.06	1.57	1.56	1.18	beta-2 microglobulin
Ccl8	cytokine	1.00	2.35	1.72	3.24	5.43	6.24	1.68	1.92	chemokine (C-C motif) ligand 8
Cxcl9	cytokine	1.00	2.13	1.88	3.02	4.39	5.34	1.45	1.77	chemokine (C-X-C motif) ligand 9
Cxcl10	cytokine	1.00	2.03	1.37	1.38	3.06	1.96	2.21	1.42	chemokine (C-X-C motif) ligand 10
Ifit1	innate antiviral/IFN	1.00	2.69	1.43	1.00	3.56	1.41	3.54	1.41	interferon-induced protein with tetratricopeptide repeats 1
Mx1	innate antiviral/IFN	1.00	1.97	1.36	0.99	2.38	1.35	2.39	1.35	MX dynamin-like GTPase 1
Mx2	innate antiviral/IFN	1.00	1.91	1.19	0.93	2.39	1.27	2.57	1.36	MX dynamin-like GTPase 2
Saa3	immune (other)	1.00	7.52	2.35	1.34	12.55	1.80	9.39	1.35	serum amyloid A 3
Col1a2	matrix	1.00	1.15	1.03	0.57	0.72	0.56	1.27	0.98	collagen, type I, alpha 2
Sparc	matrix	1.00	1.03	0.90	0.64	0.62	0.53	0.97	0.83	secreted acidic cysteine rich glycoprotein

QPCR

Symbol	TaqMan assay ID	YM0/YM0	YR2/YM0	YR5/YM0	OM0/YM0	OR2/YM0	OR5/YM0	OR2/OM0	OR5/OM0	Gene name
B2m	Mm00437762_m1	1.00	1.51	1.39	1.39	2.23	1.61	1.60	1.16	beta-2 microglobulin
Ccl8	Mm01297183_m1	1.00	4.14	3.43	8.23	17.91	15.01	2.18	1.82	chemokine (C-C motif) ligand 8
Cxcl9	Mm00434946_m1	1.00	3.23	3.98	7.05	12.10	9.14	1.71	1.30	chemokine (C-X-C motif) ligand 9
Cxcl10	Mm00445235_m1	1.00	4.76	3.28	3.53	10.26	4.40	2.90	1.24	chemokine (C-X-C motif) ligand 10
Ifit1	Mm00515153_m1	1.00	3.04	1.61	1.15	4.16	1.46	3.61	1.27	interferon-induced protein with tetratricopeptide repeats 1
Mx1	Mm00487796_m1	1.00	4.83	2.91	1.28	5.75	2.44	4.50	1.91	MX dynamin-like GTPase 1
Mx2	Mm00488995_m1	1.00	3.00	1.62	1.06	3.80	1.45	3.59	1.37	MX dynamin-like GTPase 2
Saa3	Mm00441203_m1	1.00	5.63	1.83	1.86	12.93	1.42	6.96	0.76	serum amyloid A 3
Col1a2	Mm00483888_m1	1.00	0.98	0.93	0.51	0.62	0.46	1.20	0.89	collagen, type I, alpha 2
Sparc	Mm00486332_m1	1.00	0.92	0.87	0.65	0.63	0.52	0.97	0.81	secreted acidic cysteine rich glycoprotein
